# A non-invasive, quantitative study of broadband spectral responses in human visual cortex

**DOI:** 10.1371/journal.pone.0193107

**Published:** 2018-03-12

**Authors:** Eline R. Kupers, Helena X. Wang, Kaoru Amano, Kendrick N. Kay, David J. Heeger, Jonathan Winawer

**Affiliations:** 1 Department of Psychology and Center for Neural Science, New York University, New York, New York, United States of America; 2 Center for Information and Neural Networks (CiNet), National Institute of Information and Communications Technology, Osaka, Japan; 3 Center for Magnetic Resonance Research, University of Minnesota, Minneapolis, Minnesota, United States of America; Harvard Medical School, UNITED STATES

## Abstract

Currently, non-invasive methods for studying the human brain do not routinely and reliably measure spike-rate-dependent signals, independent of responses such as hemodynamic coupling (fMRI) and subthreshold neuronal synchrony (oscillations and event-related potentials). In contrast, invasive methods—microelectrode recordings and electrocorticography (ECoG)—have recently measured broadband power elevation in field potentials (~50–200 Hz) as a proxy for locally averaged spike rates. Here, we sought to detect and quantify stimulus-related broadband responses using magnetoencephalography (MEG). Extracranial measurements like MEG and EEG have multiple global noise sources and relatively low signal-to-noise ratios; moreover high frequency artifacts from eye movements can be confounded with stimulus design and mistaken for signals originating from brain activity. For these reasons, we developed an automated denoising technique that helps reveal the broadband signal of interest. Subjects viewed 12-Hz contrast-reversing patterns in the left, right, or bilateral visual field. Sensor time series were separated into evoked (12-Hz amplitude) and broadband components (60–150 Hz). In all subjects, denoised broadband responses were reliably measured in sensors over occipital cortex, even in trials without microsaccades. The broadband pattern was stimulus-dependent, with greater power contralateral to the stimulus. Because we obtain reliable broadband estimates with short experiments (~20 minutes), and with sufficient signal-to-noise to distinguish responses to different stimuli, we conclude that MEG broadband signals, denoised with our method, offer a practical, non-invasive means for characterizing spike-rate-dependent neural activity for addressing scientific questions about human brain function.

## Introduction

The time-varying electric and magnetic fields near neural tissue provide an indirect but rich source of information about the activity of neural populations [reviewed by 1]. These signals include rapid, ‘evoked’ responses that are time-locked to stimulus events [2], oscillatory responses [3], and non-oscillatory, broadband signals [4, 5]. Broadband signals associated with sensory or motor tasks have been widely observed in human electrocorticography, or ‘ECoG’, [6] and animal microelectrode recordings [7]. The broadband signal is an elevation in spectral power, typically spanning 50 to >200 Hz [8], and has attracted a great deal of attention for several reasons.

First, the broadband signal is correlated with the level of neural activity (multi-unit spiking). This has been shown by simultaneous measures of multiunit spiking and field potentials [9, 10], as predicted by computational modeling [8, 11]. Measuring broadband power can therefore be an effective way to study population-level spiking activity in a cortical region [8–13]. Second, the broadband signal has a smaller point spread function on the cortical surface than low frequency oscillations (8–25 Hz) [5, 14], and is therefore useful both for characterizing local properties of cortex and as a tool for neural prosthetics [15]. Third, the broadband signal is correlated with a portion of the fMRI response and, together with other field potential measures, can be used to understand neural factors underlying an observed BOLD response [14, 16–18]. Finally, because it can be measured at high temporal resolution, the broadband signal is useful for characterizing the temporal dynamics of neuronal activity [19, 20]. For these reasons, broadband signals are often the primary, and sometimes only, signal of interest reported in ECoG papers.

In contrast to intracranial recordings, in the extracranial measures of electroencephalography (EEG) and magnetoencephalography (MEG), broadband responses have not been widely and reliably observed, particularly in the responses of individual sensors. One significant challenge in identifying broadband in extracranial measures is that non-neural noise sources, particularly from miniature saccades, can be confounded with experimental designs, making neurally induced broadband responses hard to isolate [21–24].

A second challenge in measuring broadband extracranially is that the response is most evident in high frequencies (> 60 Hz), and the signal amplitude at these frequencies is low. While intracranial recordings have relatively high signal-to-noise ratios (SNR) even at these higher frequencies [6], EEG and MEG do not [25]. Broadband signals can extend to lower frequencies [26, 27], but oscillatory processes in lower frequency bands often mask broadband measures in this range [5].

A third challenge is the potential confound between broadband signals and narrowband gamma oscillations. Narrowband gamma oscillations have been successfully measured with MEG and EEG, particularly in visual cortex for high contrast gratings [28–30]. The frequency range of these oscillations (30–100 Hz) overlaps the broadband range, but the narrowband and broadband signals reflect biologically different processes [6–8, 10]. The ability to measure one does not imply the ability to measure the other.

Several groups have reported high frequency signals measured extracranially [31–34]. However, because of the low SNR, the possible confound with eye movements and other artifacts, and the confusability of broadband signals with high-frequency narrowband oscillations, broadband signals are not typically reported in extracranial studies. We return to the relationship between our new measures and prior reports of high frequency extracranial measures in the Discussion (‘Prior measures of extracranial broadband and gamma band responses’).

Here, we sought to measure broadband signals quantitatively in the human brain using a non-invasive method (MEG). In order for this important, spike-dependent signal to be useful, it is necessary to measure it reliably in individual subjects, with a high SNR. A high SNR is essential if this signal will be widely used to study differences across stimuli, tasks, or groups. We developed a novel, automated MEG denoising algorithm adapted from prior fMRI work [35]. Our experiments were designed to elicit spatially localized neural responses in visual cortex, and eye movements were measured in a subset of subjects to test for possible confounds from non-neural sources.

## Material and methods

### Data acquisition

#### Subjects

Eight subjects (five females), ages 20–42 years (M = 28.4 / SD = 6.7 years) with normal or corrected-to-normal vision participated in the NYU study. An additional 4 subjects (M = 27.0 / SD = 7.4 years) participated in the same experiment at Center for Information and Neural Networks (CiNet), National Institute of Information and Communications Technology (NICT) in Osaka, Japan. Subjects provided written informed consent. The experimental protocol was in compliance with the safety guidelines for MEG research and was approved by the University Committee on Activities involving Human Subjects at New York University and by the ethics committee of the National Institute of Information and Communications Technology (NICT).

#### Display

Stimuli were generated using MATLAB (MathWorks, MA) and PsychToolbox [36, 37] on a Macintosh computer. NYU: Images were presented using an InFocus LP850 projector (Texas Instruments, Warren, NJ) with a resolution of 1024 x 768 pixels and refresh rate of 60 Hz. Images were projected via a mirror onto a front-projection translucent screen at a distance of approximately 42 cm from the subject’s eyes (field of view: 22 deg × 22 deg). The display was calibrated with the use of a LS-100 luminance meter (Konica Minolta, Singapore) and gamma-corrected using a linearized lookup table. CiNet: The display parameters were similar, except that the projector was PT-DZ680 (Panasonic, Japan), with 800 x 600 resolution and 60 Hz, and 61 cm viewing distance.

#### Stimuli

**Fig 1:**
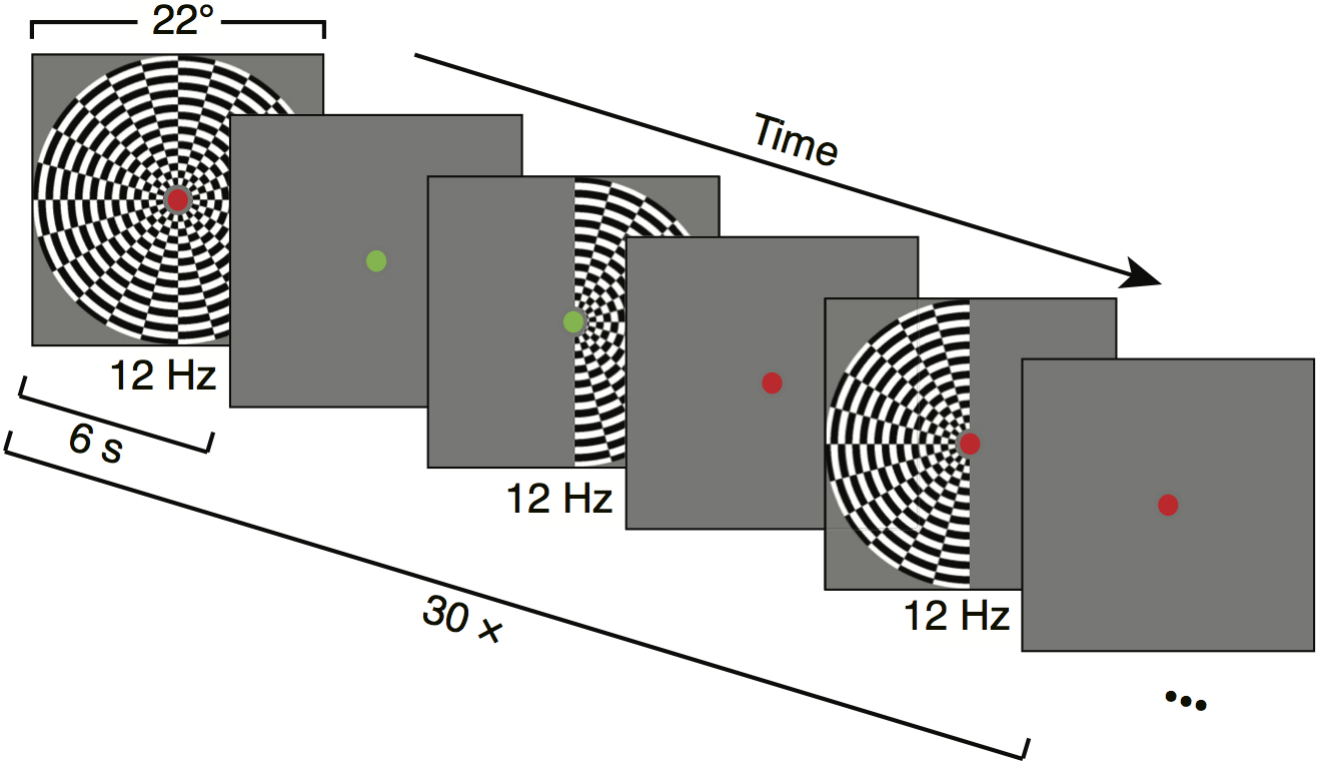
Overview of experimental design. Large-field on-off stimuli were presented in 6-s blocks consisting of either both-, left-, or right-hemifield flicker, alternating with 6-s blocks of blanks (mean luminance). A run consisted of six stimulus and six baseline blocks, after which the subject had a short break. The figure shows the first half of one run. Within a run, the order of both-, left-, and right-field flickering periods was randomized. Fifteen runs were obtained per subject, so that there were 30 repetitions of each stimulus type across the 15 runs. The fixation dot is increased in size for visibility, and shown in gray scale. Actual fixation dot was 0.17 degrees in radius (6 pixels).

The stimuli were contrast-reversing dartboard patterns (12 square wave contrast reversals per second), windowed within either a half circle (left or right visual field) or full circle (bilateral visual field) aperture, with a diameter of 22 degrees at NYU (26 degrees at CiNet). Mean luminance gray (206 cd/m^2^ (NYU), 83 cd/m^2^ (CiNet)) was used as background color for the dartboards and was shown in the full field during blank trials between stimulus periods (Fig 1).

#### Experimental design

One *run* consisted of six seconds flickering ‘on’ periods, alternated with six seconds ‘off’ mean luminance periods, repeated 6 times (72 seconds). The order of the both-, left-, or right-visual hemifield apertures was random. There was a fixation dot in the middle of the screen throughout the run, switching between red and green at random intervals (averaging 3 seconds). The subjects were instructed to maintain fixation throughout the run and press a button every time the fixation dot changed color. The subjects were asked to minimize their blinking and head movements. After every 72-second run, there was a short break (typically 30-s to 1 minute). Each subject participated in 15 runs.

#### MEG signal acquisition

Data for the main experiment were acquired continuously with a whole head Yokogawa MEG system (Kanazawa Institute of Technology, Japan) containing 157 axial gradiometer sensors to measure brain activity and 3 orthogonally-oriented reference magnetometers located in the dewar but away from the brain area, used to measure environmental noise. The magnetic fields were sampled at 1000 Hz and were filtered during acquisition between 1 Hz (high pass) and 200 Hz (low pass).

In a subset of subjects (S6-S8), eye movements were recorded by an EyeLink 1000 (SR Research Ltd., Osgoode, ON, Canada). Right eye position data were continuously recorded at a rate of 1000 Hz. Calibration and validation of the eye position was conducted by having the subject saccade to locations on a 5-point grid. Triggers sent from the presentation computer were recorded by the EyeLink acquisition computer. The same triggers were recorded simultaneously by the MEG data acquisition computer, allowing for synchronization between the eye-tracking recording and MEG recording.

The 4 data sets acquired with an Elekta Neuromag at CiNet were preprocessed in MATLAB (MathWorks, MA, USA) using the identical code and procedure. The CiNet data were acquired as 102 pairs of planar gradiometer signals (204 sensors). Data were analyzed from each of the 204 gradiometers separately and paired into 102 locations for mesh visualization (e.g., the broadband signal-to-noise ratio for sensor 121 and 122 out of 204 would be averaged to show one signal-to-noise ratio in the position of sensor 61 out of 102).

### Data analysis

#### Reproducible computation and code sharing

All analyses were conducted in MATLAB. In the interest of reproducible computational methods, both the analysis code and the MEG data for all results reported in this paper are publicly available via the Open Science Framework at the url https://osf.io/c59sh/ (doi 10.17605/OSF.IO/C59SH). Figs 2–14 (except 3) and S1–S7 Figs can be reproduced by running scripts from the GitHub repository of the form *nppMakeFigure4*.*m* (to reproduce Fig 4 from raw data), or the master script *nppMakeAllFigures*.*m*.

**Fig 2:**
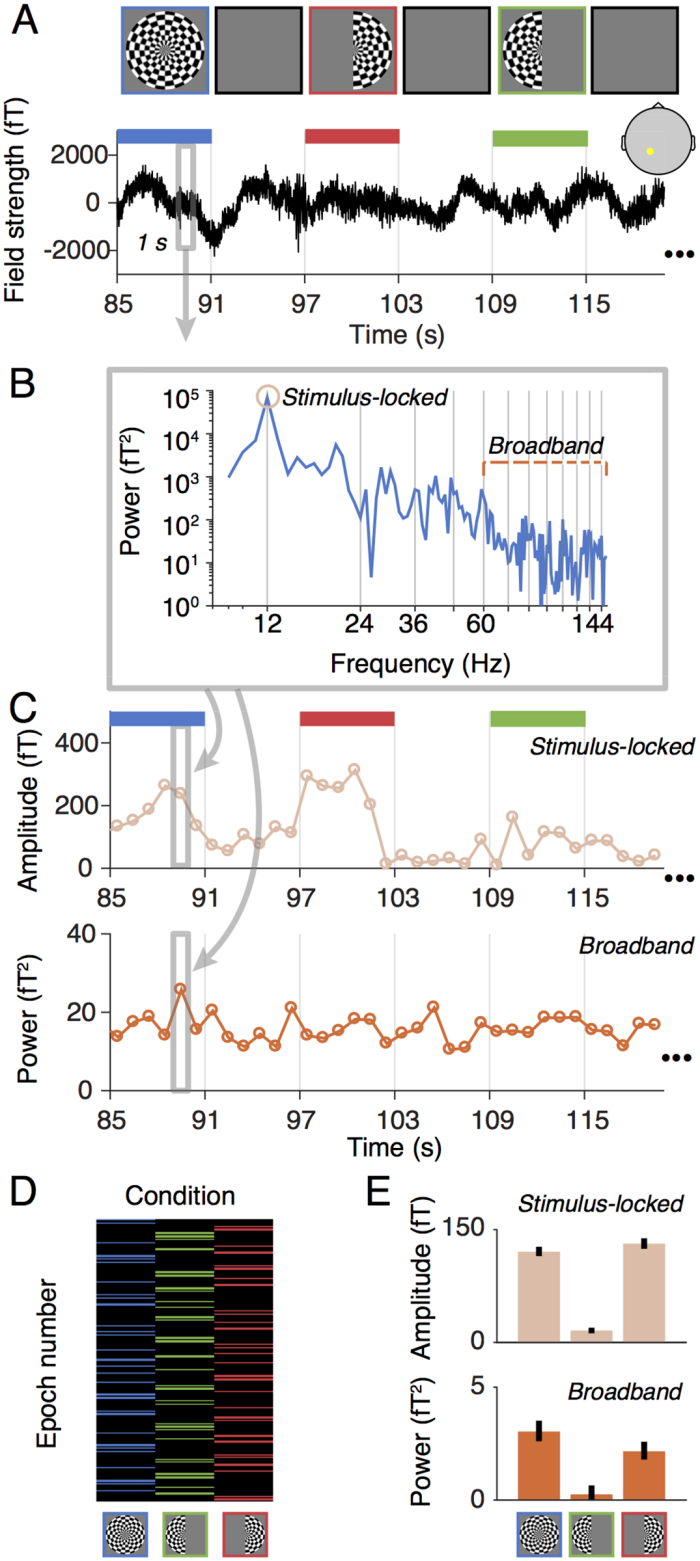
Data analysis without denoising. (A) The time series for each sensor were epoched into non-overlapping one-second periods. (B,C) The time series in each epoch was fast Fourier transformed and then summarized as two values, a stimulus-locked value (amplitude of the fast Fourier component at the stimulus frequency), and a broadband value (mean of the log power of all frequencies from 60–150 Hz, excluding those within +/- 1 Hz of stimulus harmonics). (D) The summary of conditions is shown as a matrix, where each column corresponds to one of the three stimulus conditions, and the number of rows is equal to the total number of epochs across the session. Rows with no color are blank epochs. (E) Summary metrics were computed separately for the stimulus-locked values and broadband measures, yielding three measures per sensor per data type. The summary metric was the mean across condition minus the mean across blanks, bootstrapped 1000 times. The bar plot show the mean across epochs and the standard deviation across 1000 bootstraps. Made with function *nppMakeFigure2*.*m*.

**Fig 3:**
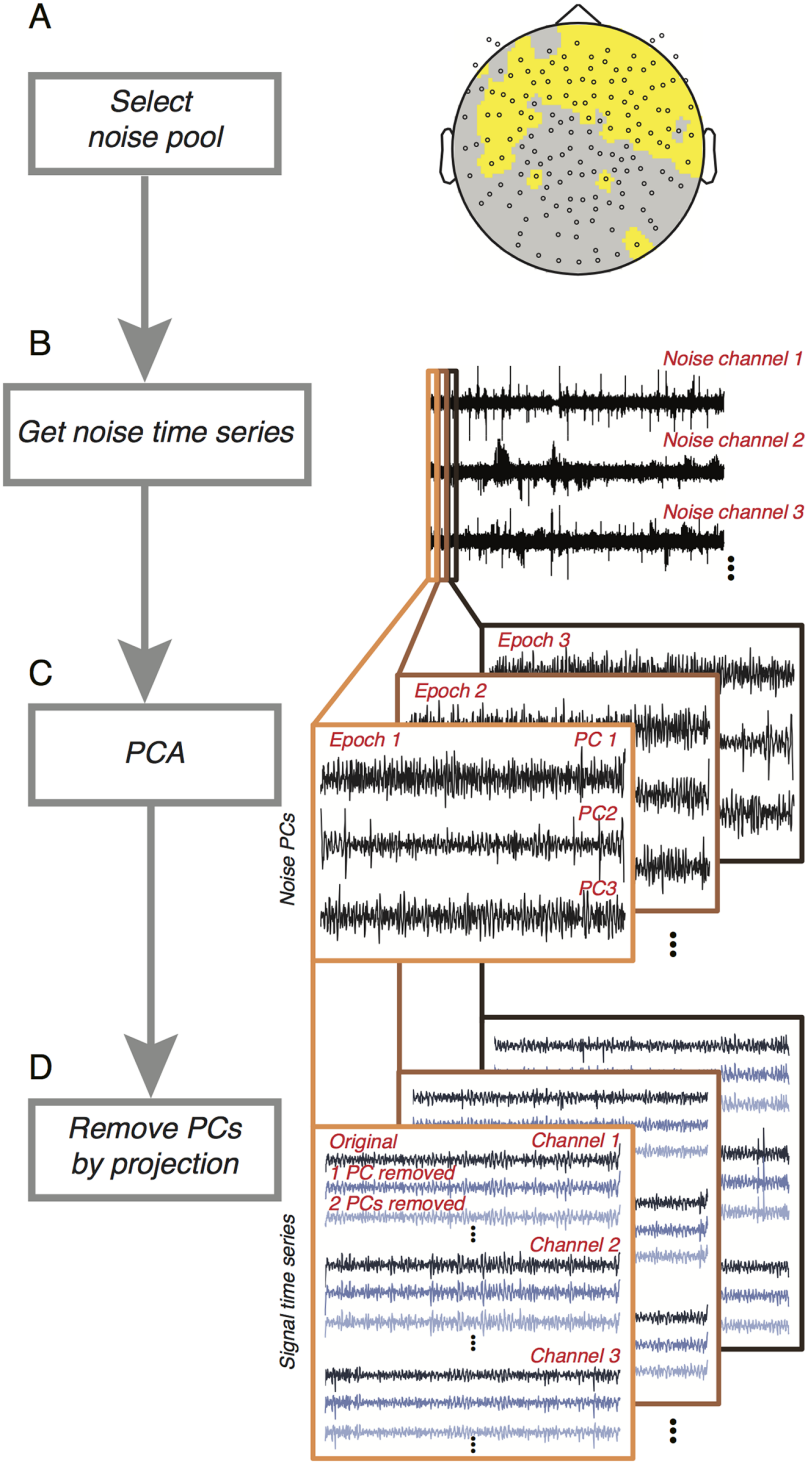
Denoising procedure. Following an estimate of response reliability computed from non-denoised data (Fig 2), the algorithm first selects a noise pool. (A) The noise pool is comprised of sensors whose SNR from the evoked (stimulus-locked) component falls below a threshold. (B) The time series from each sensor in the noise pool is then filtered to remove components that do not contribute to the broadband computation. (C) Principal component analysis is then computed within each epoch. (D) for each epoch, the first *n* PCs are projected out from the time series of all sensors, yielding *n* new data sets. For each new data set, broadband responses were recomputed, as in Fig 2.

**Fig 4:**
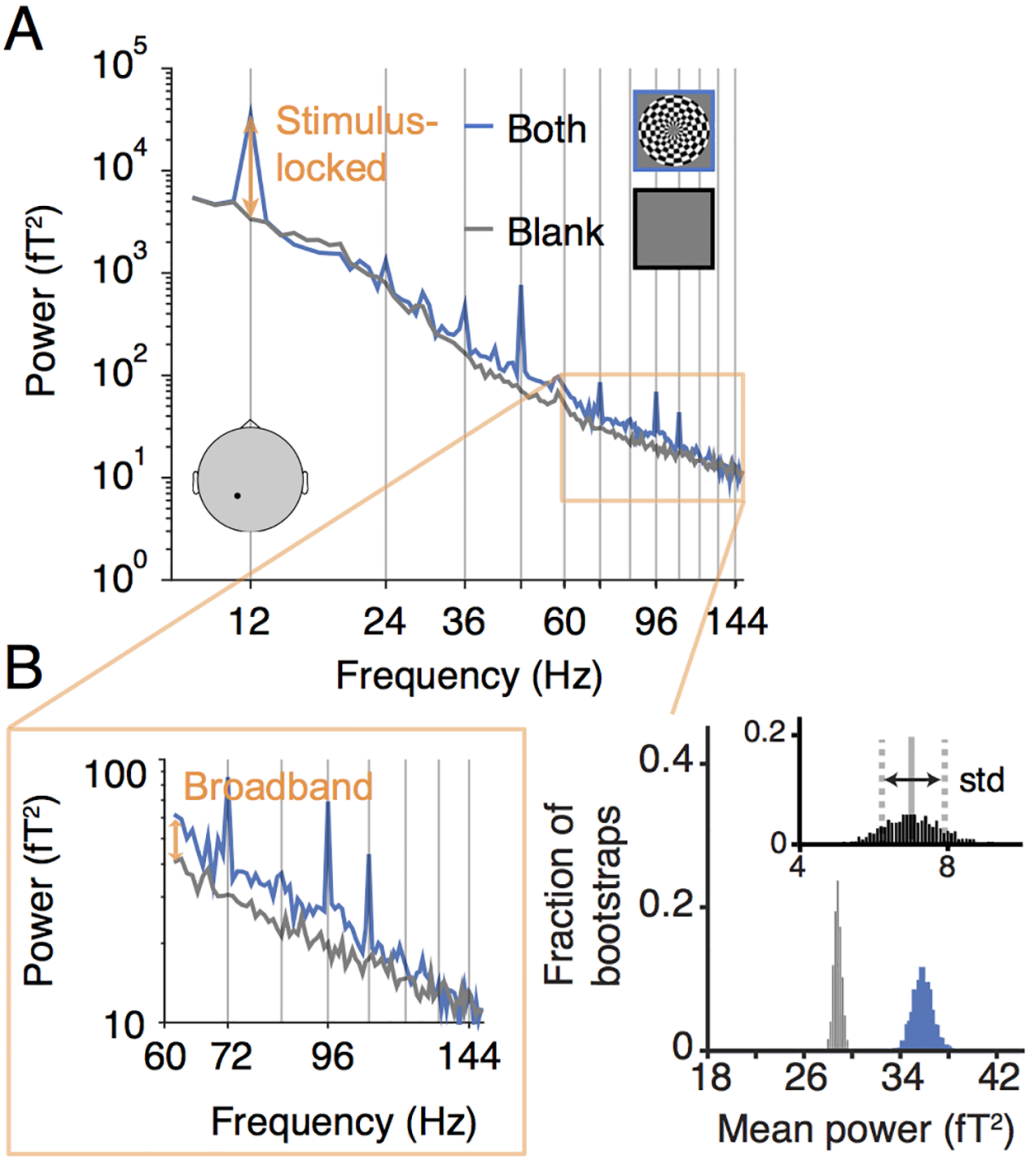
Example response to flickering both-hemifield stimulus. (A) The main panel plots the spectral power, averaged across 148 1-s epochs, during which the subject viewed either the both-hemifield stimulus (blue line) or a blank screen at mean luminance (gray line). The black dot on the schematic head indicates the location of the sensor. The peak at 12 Hz corresponds to the frequency of dartboard contrast reversals, and is a measure of the stimulus-locked component (orange arrow). (B) The lower inset zooms in on higher frequencies to emphasize the broadband component, most evident in this example data set as a spectral power elevation spanning 60 to 150 Hz. The increase in the broadband response of the stimulus condition relative to the blank condition is shown by the orange arrow. The histograms on the right show the broadband level separately for the stimulus condition (blue) and the blank condition (gray), and the difference between them (black inset), computed 1000 times by bootstrapping over epochs in the experiment. Data from subject S1. Made with function *nppMakeFigure4*.*m*.

**Fig 5:**
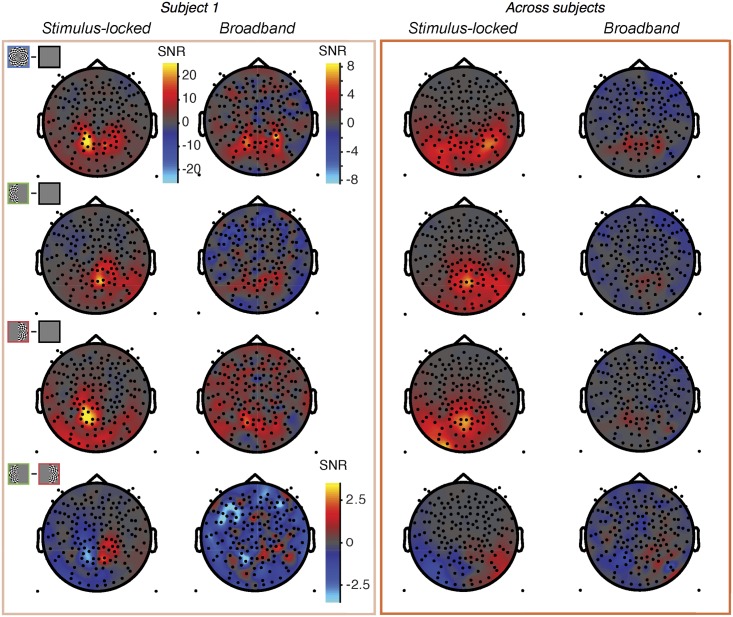
Topographic map of stimulus-locked and broadband responses. Data from subject S1 (left) and averaged across subjects S1-S8 by sensor (right). The top 3 rows show data from the 3 stimulus conditions (both-, left-, and right-hemifield) compared to blank, and the lower row shows data as the left-only minus right-only conditions. The dependent variable plotted for the single subject data is the signal-to-noise ratio at each sensor, computed as the mean of the contrast (stimulus minus blank) divided by the standard deviation across bootstraps (bootstrapped over epochs). For the group data, the signal-to-noise ratio is the mean of the subject-specific SNRs at each given sensor. The same scale bar is used for all stimulus-locked plots. For the broadband plots, one scale bar is used for the first three rows, and a different scale bar with a smaller range is used for the fourth row. Made with *nppMakeFigure5*.*m*.

**Fig 6:**
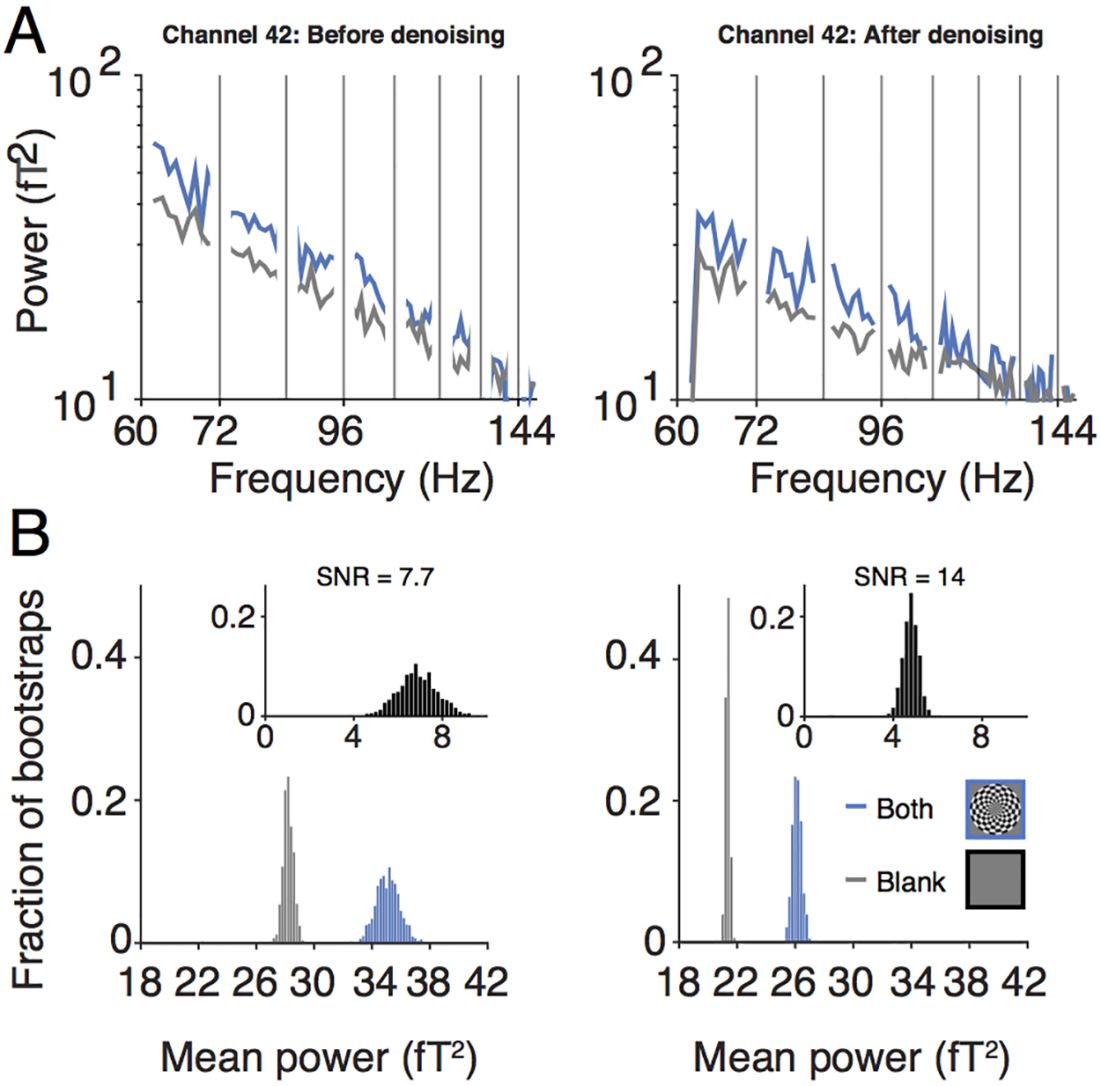
Effect of denoising on broadband response. (A) The upper panel shows the power spectra from sensor 42, subject S1, averaged across 178 epochs with the both-hemifield stimulus (blue) and blank screen (gray). The left panel is prior to denoising and is identical to the inset in Fig 4, except that harmonics of stimulus-locked frequencies have been removed. The right panel is the same as the left, except after denoising. (B) The lower panel shows the distributions of the bootstrapped broadband power for the both-hemifield (blue), blank (gray), and both-hemifield minus blank (black, inset), prior to denoising (left) and after denoising (right). The SNR is defined as the mean of the difference between both-hemifield and blank epochs divided by the standard deviation across bootstraps of the difference distribution (7.7 prior to denoising, 14.0 after). The effects of denoising are to reduce the mean power, and more importantly, reduce the standard deviation across epochs. Made with function *nppMakeFigure6*.*m*.

**Fig 7:**
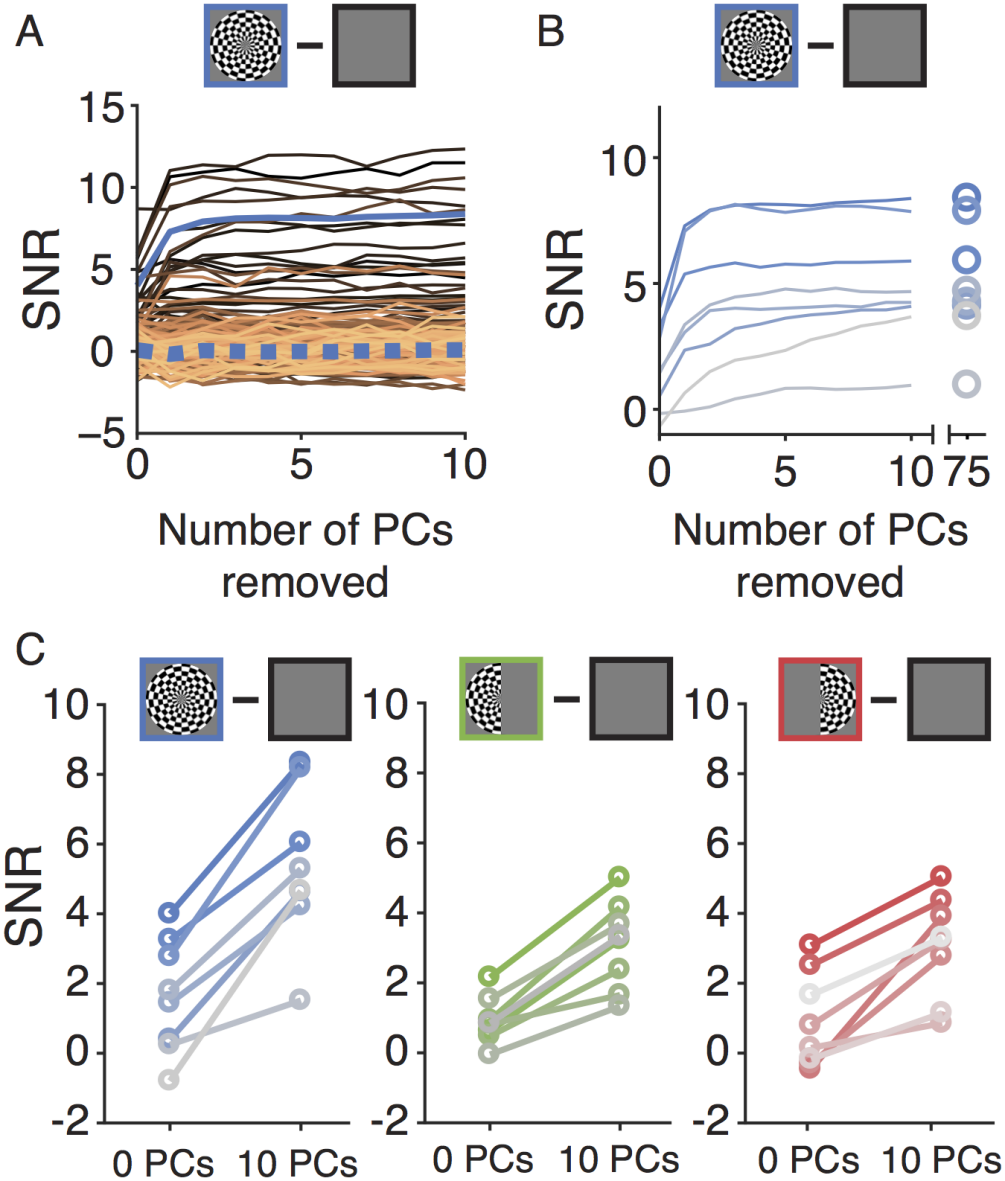
Effect of denoising on broadband SNR. (A) SNR as a function of the number of PCs projected out in subject S1 for the both-hemifield stimulus. Each line is one sensor. The solid blue line is the mean of the 10 sensors of interest, chosen as those sensors with the highest SNR, as measured either before or after denoising (see ‘Statistical comparisons’ in Material and methods). The dotted blue line is the mean of the 75 sensors in the noise pool, as measured either before or after denoising. (B) SNR as a function of PCs projected out in each of 8 subjects for the both-hemifield stimulus. Each line is the mean across the 10 sensors with the highest SNR in one subject. The rightmost points indicate the effect of projecting out all 75 PCs. (C) SNR before denoising (0 PCs projected out) and after denoising (10 PCs projected out) for each stimulus condition. Each line is the mean of the 10 sensors with the highest SNR for one subject in one stimulus condition. Color saturation corresponds to the subject number (highest to lowest saturation, subjects 1–8, respectively). Made with function *nppMakeFigure7*.*m*.

**Fig 8:**
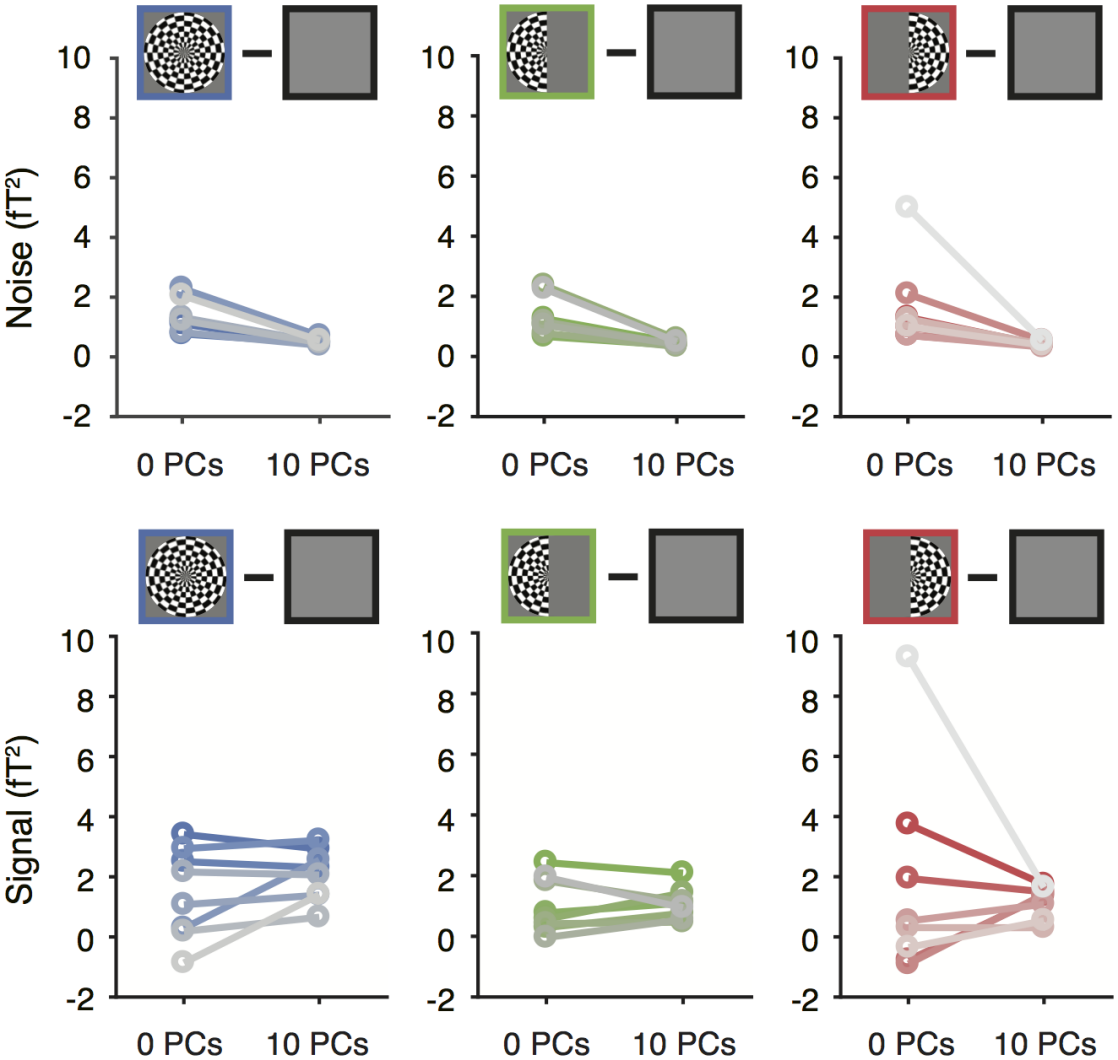
Effect of denoising on the broadband signal and noise. Noise (upper) and signal (lower) before and after denoising in each of three stimulus conditions. Plotting conventions as in Fig 7c. Made with function *nppMakeFigure8*.*m*.

**Fig 9:**
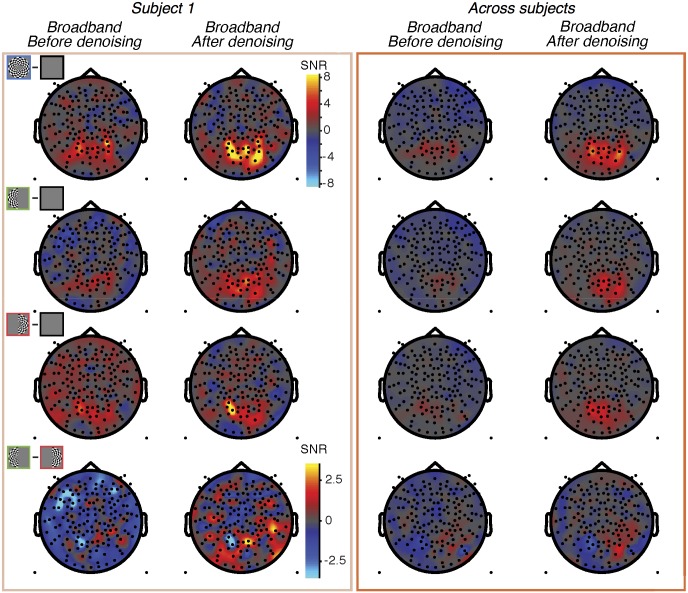
Topographic map of broadband SNR before and after denoising. Data from subject S1 (left) and averaged across subjects S1-S8 by sensor (right). The top 3 rows show data from the 3 stimulus conditions (both-, left-, and right-hemifield) and the fourth row shows the difference between the left-only and right-only conditions. The fourth row uses a different scale bar from the other 3 rows. The columns show data before and after denoising. Made with function *nppMakeFigure9*.*m*.

**Fig 10:**
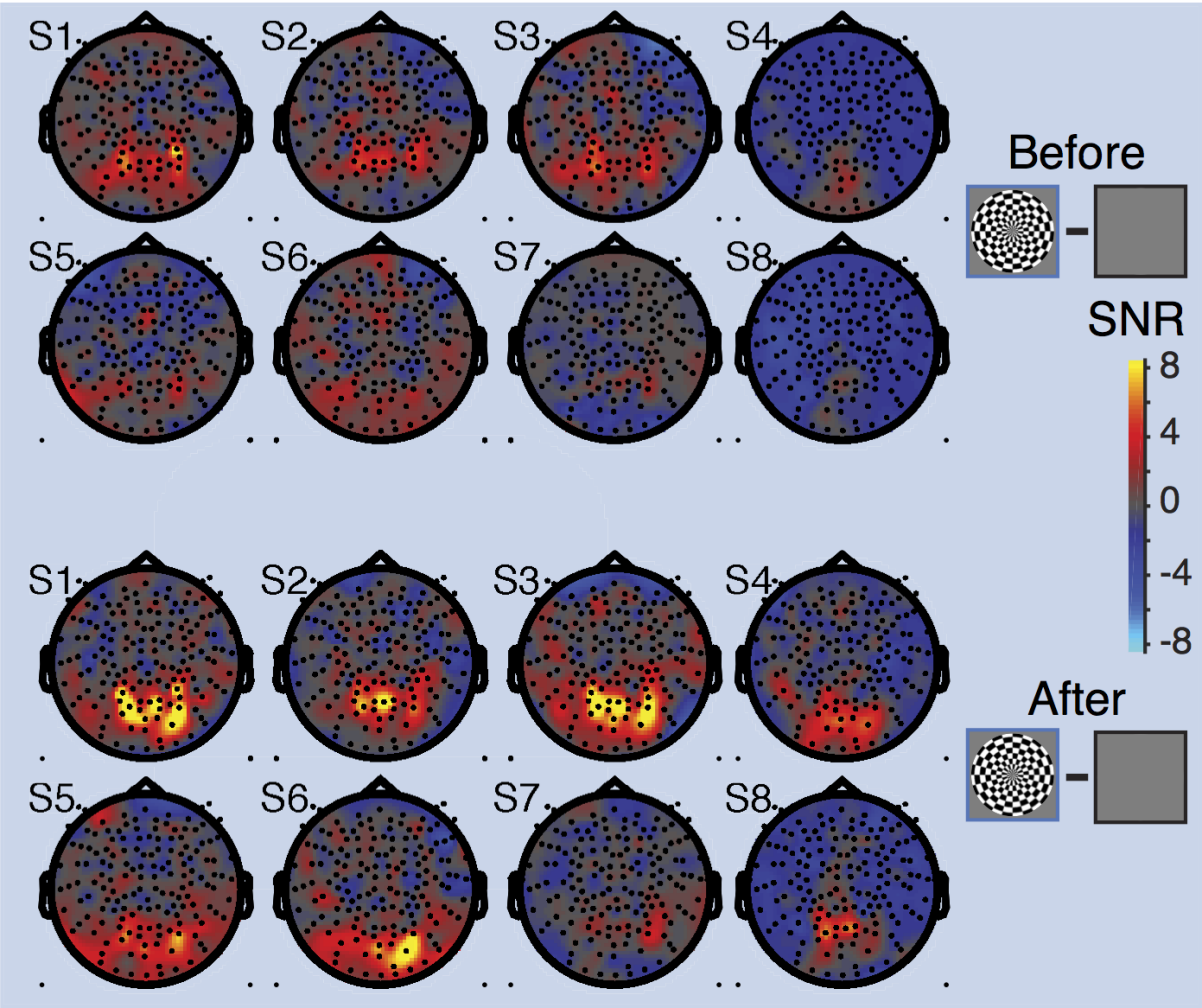
Topographic maps of broadband SNR in individual subjects after denoising. Head plots show the SNR for the both-hemifield stimulus, before denoising (above) and after denoising (below). For individual subject data for all stimulus conditions, see S6 Fig. Made with function *nppMakeFigure10*.*m*.

**Fig 11:**
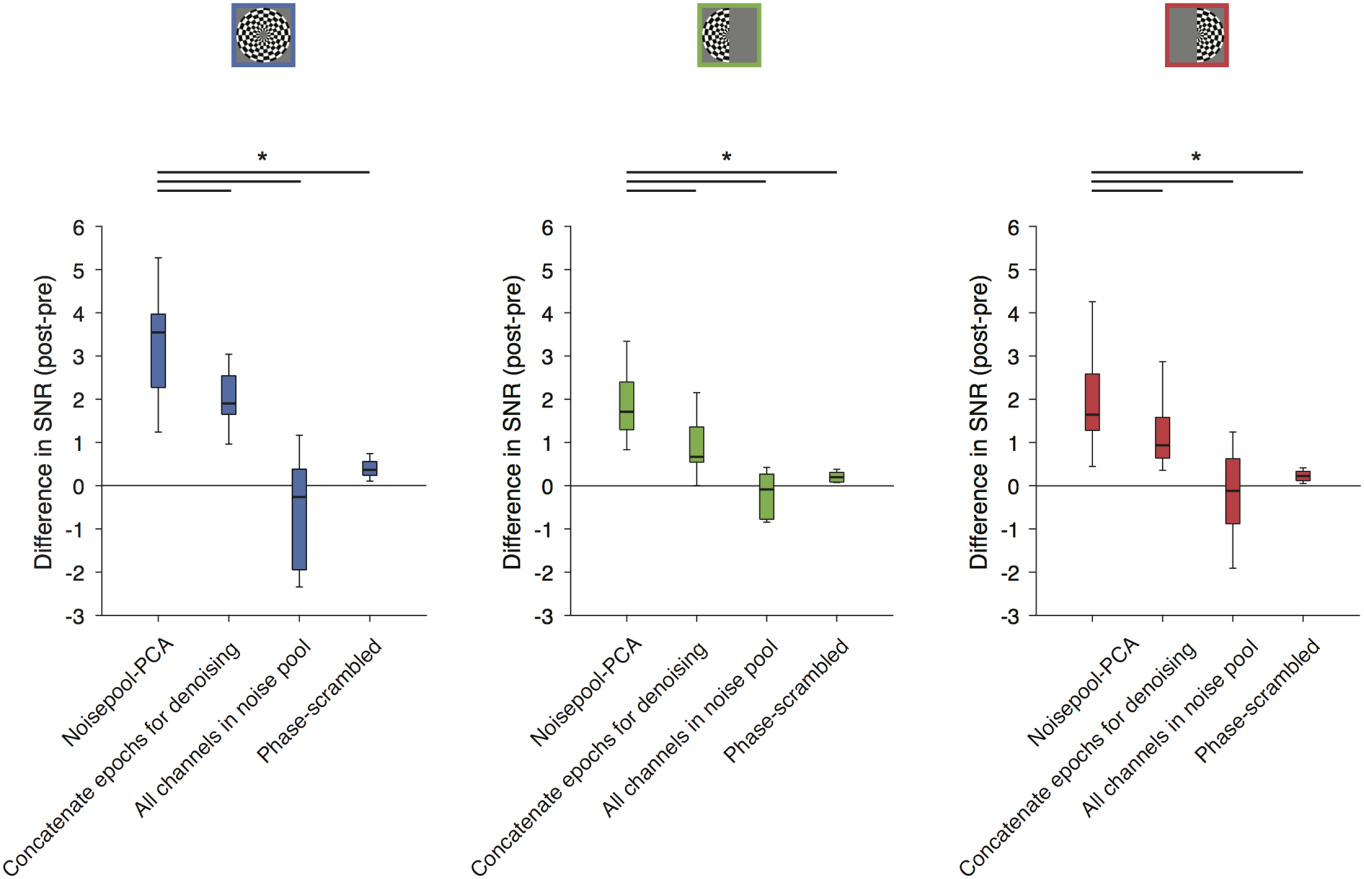
Comparison of Noisepool-PCA to control analyses. Boxplots show the difference in SNR for each stimulus condition using the Noisepool-PCA algorithm and 3 control algorithms. The black bar indicates the median, the boxes are one quartile above and below the median, and the bars are the range. When the denoising algorithm derives noise regressors from the whole experimental time series (‘Concatenate epochs for denoising’), the amount of SNR gain is significantly less than the standard Noisepool-PCA (regressors derived separately from each 1-s epoch). When the noise regressors are derived from all sensors (‘All sensors in noise pool’), or when the time series of the regressors are phase-scrambled, there is little or no change in SNR for all three stimulus conditions. Statistical significance was computed by a paired t-test between SNR values for Noisepool-PCA and other denoising analyses, paired by subject (see Material and methods). Statistical significance is indicated by * = *p* < 0.05 between the Noisepool-PCA algorithm and each of the other the algorithms. P-values are two-tailed. Made with function *nppMakeFigure11*.*m*.

**Fig 12:**
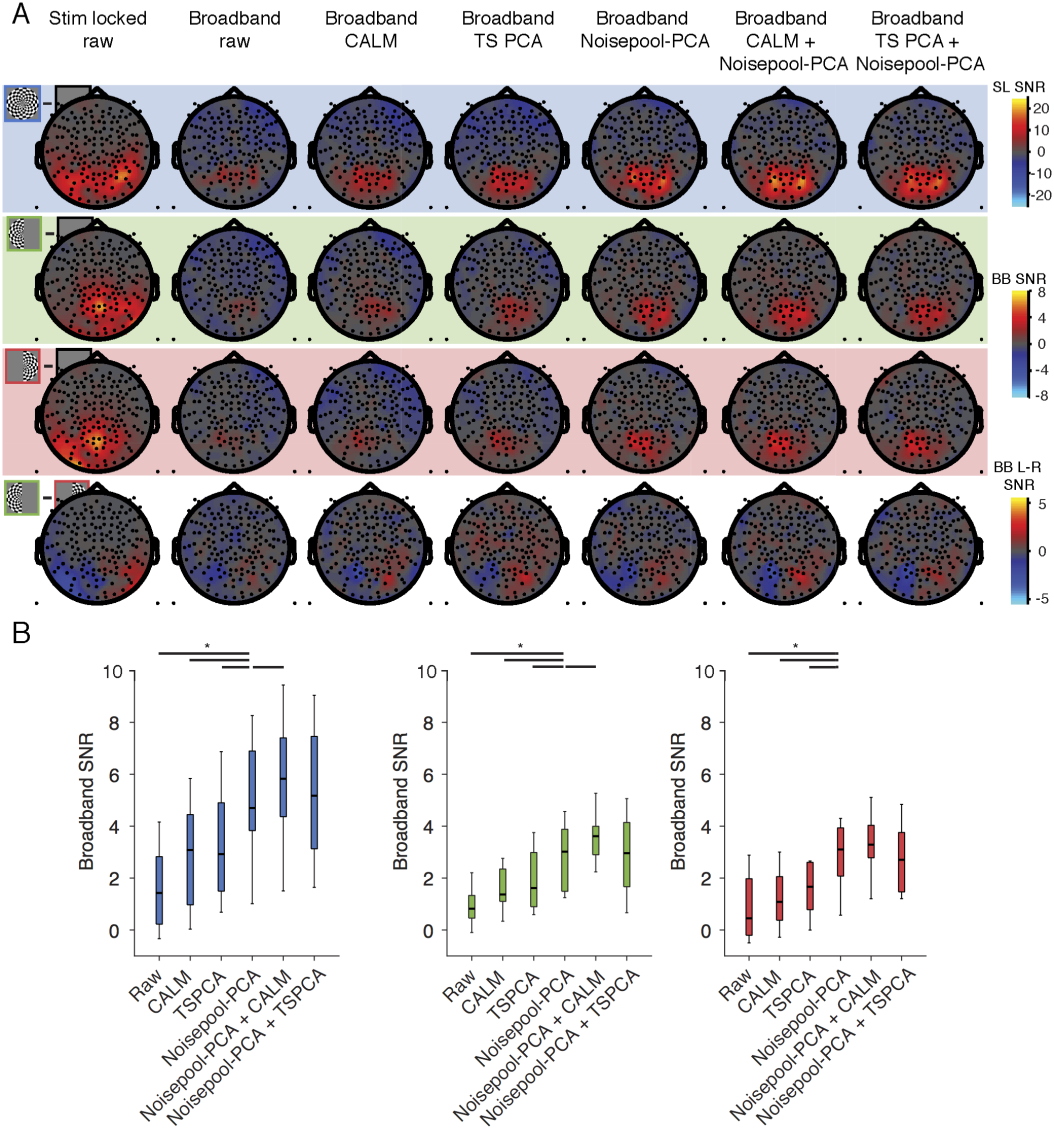
Comparison of different denoising algorithms on NYU datasets (averaged across subjects S1-S8). (A) The columns represent SNR values for the stimulus-locked signal (column 1), broadband signal without denoising (column 2), and broadband signal with one or more denoising algorithms. One scale bar is used for all stimulus-locked plots (column 1). A second scale bar is used for all broadband plots (columns 2–7) except for the left minus right plots (row 4, columns 2–7). Other details as in Fig 5. (B) Broadband SNR using different algorithms for both-hemifield (left), left-hemifield (center) and right-hemifield (right) stimuli. Each boxplot is the change in SNR from baseline (column 2 in panel A), averaged across the top 10 sensors per subject). Top sensors were defined as the 10 sensors from each subject with the highest SNR across any of the 3 stimulus conditions and any of the denoising algorithms (columns 2–7). The boxplots show the median (black horizontal line), quartiles (boxes), and total range (error bars) across subjects. Statistical significance was computed by paired-tests, as in Fig 11. Statistical significance is indicated by * = *p* < 0.05 between the Noisepool-PCA algorithm and each of the other the algorithms. P-values are two-tailed. Made with function *nppMakeFigure12*.*m*.

**Fig 13:**
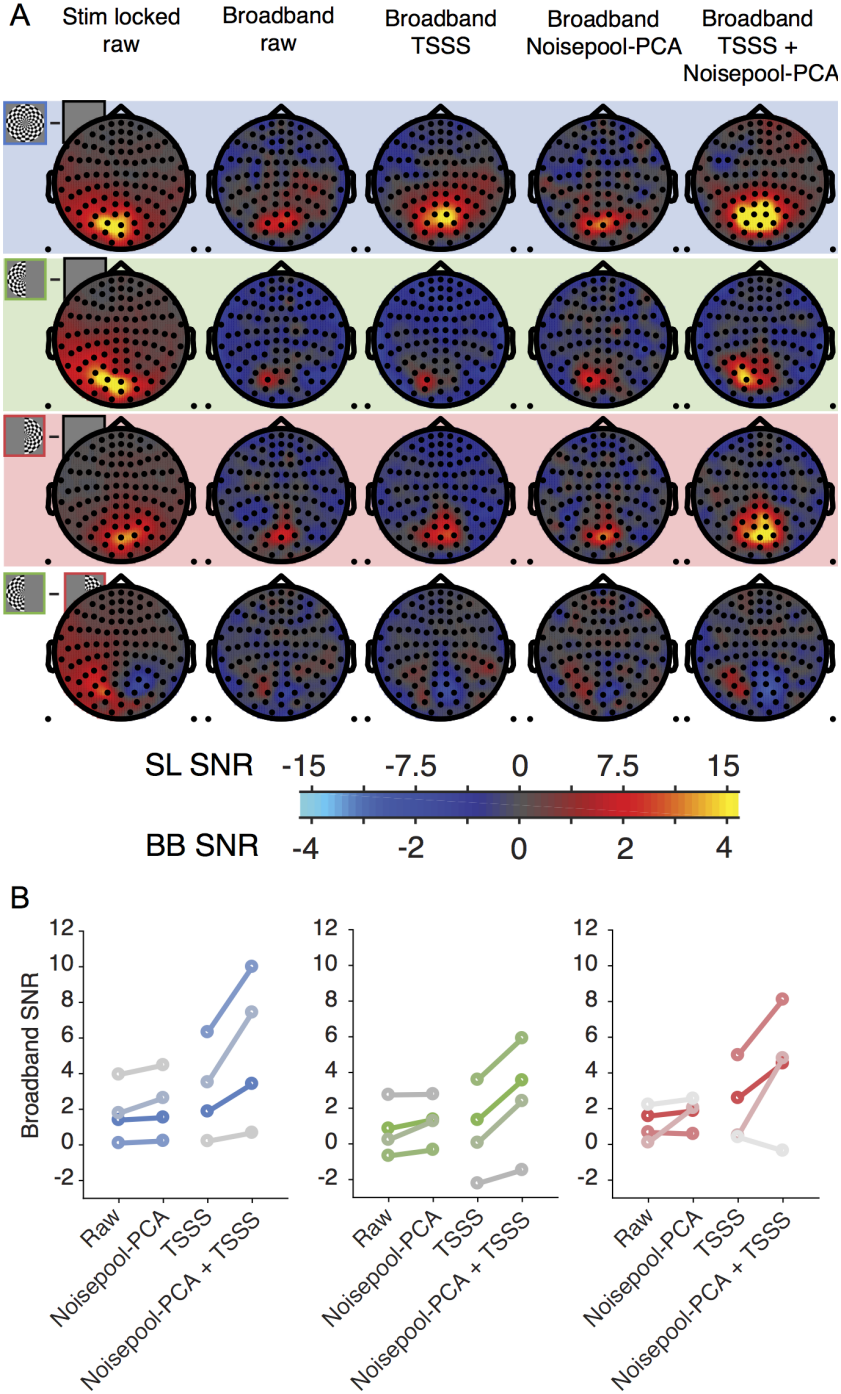
MEG data from CiNet Neuromag. (A) All plots show data averaged across new 4 subjects (S9-S13) in sensor space (sensor-wise mean of the subject SNR). The columns represent SNR values for the stimulus-locked signal (column 1), broadband signal without denoising (column 2), and broadband signal with one or more denoising algorithms. The same scale bar is used for all broadband data (columns 3–5). Other details as in Fig 5. (B) Broadband SNR using different algorithms for both-hemifield (left), left-hemifield (center) and right-hemifield (right) stimuli. Each line is average broadband SNR across the top 10 sensors for one individual. Top sensors were defined as the 10 sensors from each subject with the highest SNR across any of the 3 stimulus conditions and any of the denoising algorithms (columns 2–5). Made with function *nppMakeFigure13*.*m*.

**Fig 14:**
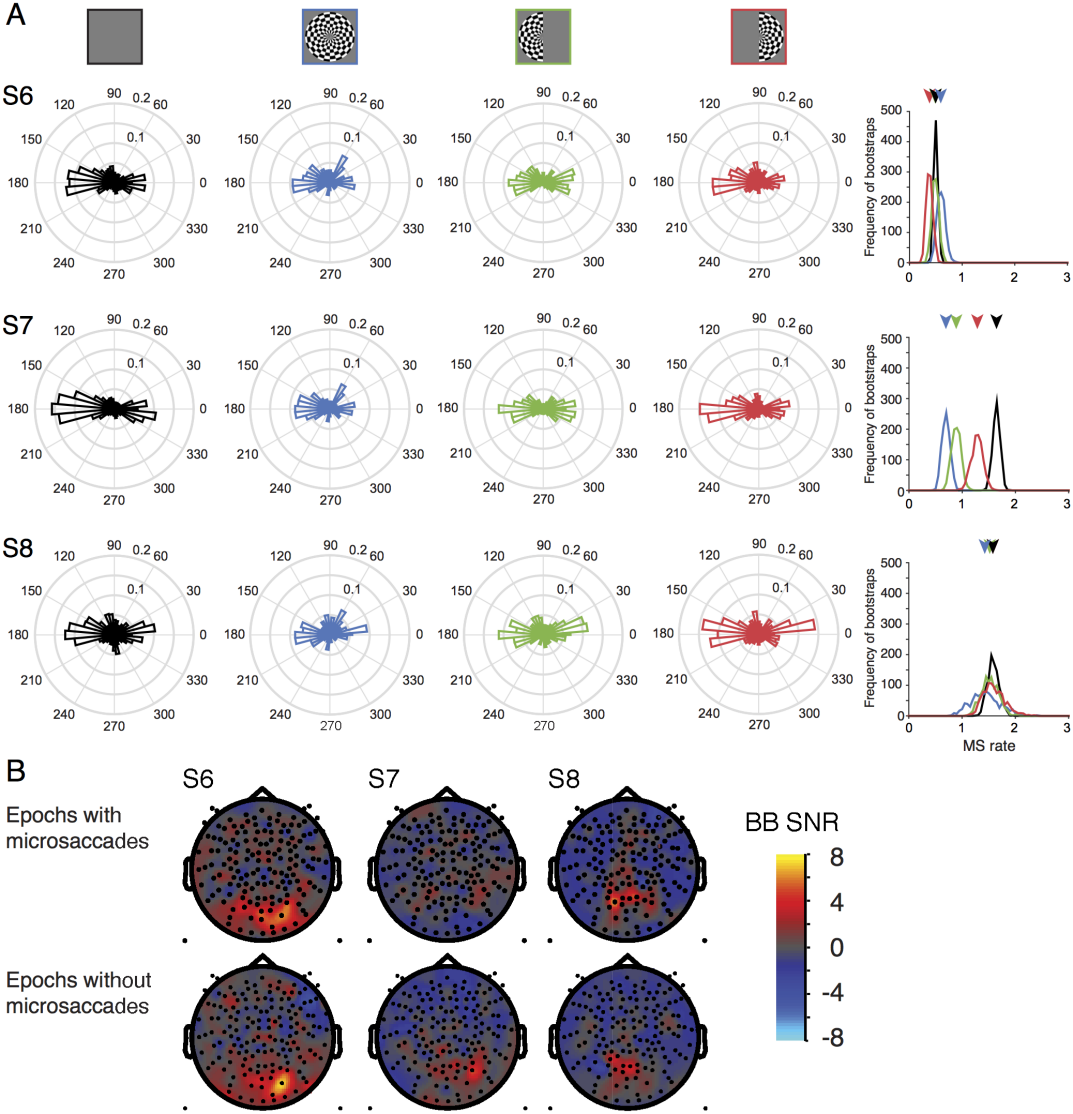
Microsaccades during experimental conditions. (A) The circular histograms show the frequency of microsaccades per 1-s epoch, binned by direction, for each of the 4 stimulus conditions (columns 1–4). The rows show data for 3 subjects. The last column shows the rate of microsaccades (per 1-s epoch) irrespective of direction, for each of the 4 stimulus conditions, bootstrapped 100 times over epochs. Arrows indicate the median rate for each condition. (B) Both-hemifield minus blank broadband SNR meshes limited to only those epochs with microsaccades (top row) or without microsaccades (bottom row). Made with function *nppMakeFigure14*.*m*.

#### MEG preprocessing

For some analyses, data were environmentally denoised using published algorithms prior to any further analysis. This enabled us to compare data denoised with our new algorithm alone, or with our new algorithm following environmental denoising. For the NYU data, we used either of two algorithms. One was the continuously adjusted least-square method (CALM; [38], applied to data with a block length of 20 seconds (20,000 time samples). The second algorithm was time-shifted principal component analysis (TSPCA; [39], with a block length of 20 seconds and shifts of up to +/- 100 ms in 1 ms steps. For the CiNet data, the environmental denoising algorithm was temporal signal space separation (‘tSSS’, [40, 41]) (with default parameters, e.g. inside and outside expansion orders of 8 and 3, respectively; 80 inside and 15 outside harmonic terms; correlation limit of 0.98).

The FieldTrip toolbox [42] was used to read the data files (either environmentally-denoised or raw). For all subsequent analyses, custom code was written in MATLAB. Using either the environmentally-denoised data or raw data, the signals were divided into short epochs. Each stimulus type (both-, left-, or right-hemifield, or blank) was presented in 6-s blocks, and these blocks were divided into 6 non-overlapping 1-s epochs. We discarded the first epoch of each 6-s block to avoid the transient response associated with the change in stimulus. After epoching the data, we used a simple algorithm to detect outliers. We first defined a ‘data block’ as the 1-s time series from one epoch for one sensor. So a typical experiment consisted of ~170,000 data blocks (157 sensors x 1080 1-s epochs). We computed the standard deviation of the time series within each data block, and labeled a block as ‘bad’ if its standard deviation was more than 20 times smaller or 20 times larger than the median standard deviation across all data blocks. The time series for bad data blocks were replaced by the time series spatially interpolated across nearby sensor (weighting sensors inversely with the distance). Further, if more than 20% of data blocks were labeled bad for any sensor, then we removed the entire sensor from analysis, and if more than 20% of data blocks were bad for any epoch, then we removed the entire epoch from analysis. Typically, two to seven sensors and 2%-4% of the epochs were removed per session for the NYU data. For the CiNet datasets, almost no sensors or epochs were removed (one sensor and one epoch across all data sets). These preprocessing steps were implemented with the function *nppPreprocessData*.*m*.

#### Computation of stimulus-locked and broadband responses

Data were summarized as two values per sensor and per epoch: a stimulus-locked and a broadband power value. These calculations were done by first computing the Fourier transform of the time series within each epoch (Fig 2A and 2B).

The stimulus-locked signal was then defined as the amplitude at the stimulus-locked frequency (12 Hz). The broadband response was computed as the geometric mean of the power across frequencies within the range of 60–150 Hz, excluding multiples of the stimulus-locked frequency (see also Fig 2A and 2B). The geometric mean is the exponential of the average of the log of the signal. We averaged in the log domain because log power is better approximated by a normal distribution than is power, which is highly skewed. These two calculations converted the MEG measurements into a broadband and a stimulus-locked summary metric, each sampled once per second (Fig 2C). The two summary metrics were computed by the functions *getstimlocked*.*m* and *getbroadband*.*m*.

We then bootstrapped across epochs to compute confidence intervals on the signal estimates (per sensor and per condition). For each of 1000 bootstraps, we sampled *n* epochs with replacement, where *n* is the total number of epochs in the experiment. We then computed the average response across epochs for each stimulus condition, minus the average across blank epochs. This provided one summary measure for each of the three stimulus conditions and each of the two dependent measures (broadband and stimulus-locked) for each of the 1000 bootstraps. Finally, we took the mean of the summary measure across epochs (i.e., the sample mean) as the estimate of signal and the standard deviation across bootstraps (i.e., standard error of the mean) as a measure of variability (Fig 2D and 2E). Because the standard deviation across bootstraps is potentially a biased measure of variability for some distributions, we checked whether this was the case in our data: we compared the standard error computed this way (standard deviation of the bootstrapped mean) to half of the 68 percent confidence intervals from the bootstrapped distribution, and found the two measures to be nearly identical (*r*^*2*^ > 0.99 across bootstraps and stimulus conditions for an example subject; data can be found at Open Science Framework url https://osf.io/c59sh).

#### Noisepool-PCA algorithm

Extracranial measurements like MEG have multiple global noise sources and a relatively low signal-to-noise ratio compared to intracranial measures, especially for high frequency signals. In order to increase the signal-to-noise ratio, we developed a denoising technique that helps reveal the broadband signal of interest. A denoising algorithm developed for fMRI (‘GLMdenoise’; [35]) was adapted for MEG to project out noise from the data for each epoch in each sensor. The logic behind the algorithm is that many sources of noise are global, and therefore spread across sensors. The algorithm identifies sensors that have no stimulus-related response (the ‘noise pool’), and uses these sensors to define noise components. The noise components are then projected out from all sensor time series in each epoch.

An important difference between fMRI and MEG is that with fMRI, one is likely to obtain measurements from many parts of the brain which contain no stimulus-related response. In contrast, MEG measures sources which propagate broadly across the head due to volume conduction, and thus there is the possibility that it will not be possible to identify a pure noise pool, i.e. a set of sensors that carry no stimulus-related responses. In this case, there is the possibility that a putative noise pool will in fact contain stimulus-related signal, and that this signal will be projected out of our sensors, hence reducing signal at least as much as we reduce noise. Whether or not this is an actual problem in practice is an empirical question, and we return to it in the Results. To preview, we find that the noise pool in fact contained little to no signal, and that the diffuse spread of signals across all MEG signals was not a problem for our denoising algorithm.

#### Metric to quantify the effect of denoising

To assess the reliability of broadband responses, both before and after applying our denoising algorithm, we summarized the responses in terms of a signal-to-noise ratio (SNR). We defined SNR as the ratio of the estimated mean broadband response to the standard error, as described above (‘Computation of stimulus-locked and broadband responses’). This definition of SNR reflects the stability (but not the magnitude) of the estimated stimulus driven broadband response, and is similar to the SNR metric used for assessing the related fMRI denoising algorithm, GLMdenoise [35]. We are interested in the stability of the estimated broadband response level because the ability to distinguish the response level for different stimuli or experimental conditions is essential for addressing many scientific questions.

#### Noise pool selection

The noise pool was defined as the 75 (NYU) or 100 (CiNet) sensors with the lowest stimulus-locked SNR across conditions. The SNR was computed by (a) dividing the mean response from the data by the standard deviation across bootstraps for each condition, and (b) taking the maximum of the three values (corresponding to the three stimulus conditions) for each sensor.

We used the stimulus-locked signal to identify the noise pool because this signal had a very high SNR, and could easily by measured prior to running our Noisepool-PCA algorithm, and because we assumed (and confirmed by inspection) that sensors with broadband responses also had stimulus-locked responses. For most subjects, most of the sensors in the noise pool were located over the front of the head (see for example Fig 3A).

#### Filtering of time series

As described above, the broadband summary metric was derived from power at a limited range of temporal frequencies (60–150 Hz, excluding multiples of the stimulus frequency). After defining the noise pool, the time series of all sensors in all epochs were filtered to remove signal at all frequencies not used to compute the broadband signal. Hence the remaining time series contained power only at frequencies defining the signal of interest. This step was important because the noise pool, though selected for a low stimulus-locked SNR, could nonetheless have contained a small, residual stimulus-locked signal. This residual signal would have been correlated with the experimental design (larger when stimuli were present than absent) and hence projecting it out of the data could have caused a systematic bias (see the script *denoisingProjectingInVariance*.*m*).

#### PCA

Following filtering, the next step in the algorithm was principal component analysis (PCA). This identified the common components of the time series across the sensors in the noise pool. PCA was computed separately for each 1-s epoch (Fig 3C). This means that denoising occurred at the same temporal scale (1-s) as the computation of the summary metrics. This differs from some denoising algorithms, in which noise regressors are identified over a much longer time period, e.g., several minutes [43]. Denoising at a short-time scale can be advantageous if the spatial pattern of the noise responses is not consistent across the entire experiment. As a control comparison, we also ran our algorithm by identifying PC time series on the entire duration of the experiment (~20 minutes) rather than epoch by epoch. (See Results, ‘Control analyses for Noisepool-PCA algorithm’.) Because the PCs were computed independently for each short epoch, many thousands of PCs were computed per experiment (~1,000 epochs times 10 PCs per epoch). Hence we did not try to determine whether the temporal structure of the PCs corresponded to specific sources of noise. A statistical summary of the PC time series and spectral power is shown in S3 Fig.

#### Projecting out PCA components

The first one to ten principal components (PCs) in each epoch were projected out of the time series for all sensors, using linear regression. This resulted in ten new data sets: One with PC 1 projected out, one with PC 1 and 2 projected out, etc. up to 10 PCs projected out (Fig 3D). After projecting out the noise components, we summarized the data into a stimulus-locked and broadband component as described in Fig 2.

#### Choice of denoising parameters

We conducted several control analyses to probe how choices made in applying the denoising algorithm affected the results of denoising (amount of SNR gained). In particular, we systematically varied the number of sensors in the noise pool, the number of PCs projected out, and the number of epochs denoised at a time (S1 and S2 Figs).

#### Synthetic dataset

To illustrate how the denoising algorithm works on a known signal, we generated two synthetic data sets and analyzed them with the same code used to analyze the MEG data (S4 Fig). The first synthetic data set was comprised of a mixture of 4 components in each of 157 sensors across 30 epochs (1-s each, with ms sampling). The 4 components were stimulus-locked, broadband, background local noise (uncorrelated across sensors), and background global noise (correlated across sensors). Because the data set contains a mixture of correlated (global) and uncorrelated (local) noise, it provides a test of whether the algorithm can identify and remove the global noise sources. We defined all sensors in the back half of the mesh as responsive to the stimulus (‘visual sensors’) and the all sensors in the front half as not responsive (‘non-visual’), implemented in the following way. The stimulus-locked component was a sinusoid, present in the visual sensors during stimulus epochs and absent during blanks. This component was identical across all visual sensors. The broadband component was pink noise (1/f), present in the visual sensors and absent in the non-visual sensors. The pink noise comprising the broadband component was defined independently for each visual sensor, so it was uncorrelated across sensors. The background local noise component was also pink noise, but unlike the broadband component, it was present in all epochs (stimulus and blank) and all sensors (visual and non-visual). This component was uncorrelated across sensors. Finally, the background global noise component was a weighted sum of 10 basis time series. The weights for the different sensors were random but constrained to have a norm of 1. These time series of the 10 basis functions were independent of each other. Because this component was a weighted sum of the same 10 basis functions, this component was correlated across sensors. The expectation was that the PCA from the denoising algorithm would identify and remove the global noise, thereby increasing the SNR of the estimated broadband response. The second synthetic dataset was generated without local broadband (i.e. setting the response amplitude to zero), while keeping the stimulus-locked, uncorrelated and correlated noise components. Having a synthetic data set with a robust stimulus-locked and noise component allowed us to check whether the algorithm injects artefactual broadband signals when none is present.

#### Statistical comparisons

To assess the effect of the Noisepool-PCA algorithm on the broadband SNR, we compared the broadband SNR after applying Noisepool-PCA to the broadband SNR either without denoising or after applying other denoising algorithms. To make these comparisons, we summarized the results with one number per subject per stimulus condition in the following way. We first identified 10 sensors of interest from each subject. These 10 sensors of interest were those with the highest SNR in any of the three stimulus conditions, either before or after denoising, excluding sensors from the noise pool. For each of the three stimulus conditions, we then took the average SNR from these 10 sensors without denoising or after applying Noisepool-PCA or another denoising algorithm. Finally, we computed a two-tailed *p*-value by a paired t-test, with the pairing between analysis methods.

#### Control analyses

To investigate the validity of our algorithm, we ran multiple control analyses. In particular, it is important to rule out the possibility that the denoising algorithm produces significant results even when the data contains no sensible signal. To test this, we compared the difference in SNR of denoised data with the following controls: (1) phase-scrambling the PC time series, and (2) using all sensors to define the noise with PCA rather than only a subset of sensors that have little to no stimulus-locked signal. We also assessed the effect of identifying and projecting out PC time series equal in length to the entire experiment (~20 minutes), rather than PC time series matched in length to our analysis epochs (1-s). This comparison tested the assumption that denoising in shorter epochs was advantageous, possibly due to the pattern of noise sources differing over the course of the experiment.

#### Eye tracking analysis

Since an increase in microsaccade rate can induce broadband spectral components in extracranial measurements such as EEG or MEG [24, 44], we checked in three NYU subjects (S6-S8) whether there was a difference in rate between the ‘off’ baseline periods and ‘on’ stimulus periods, and within the three stimulus (both-, left-, right-hemifield) conditions. Microsaccades were identified as changes in position with above a relative velocity threshold (6°/s) and a minimum duration of 6 ms, as reported in Engbert & Mergenthaler [45] to analyze rate and direction of microsaccades as well as separating MEG data into epochs that did and did not contain microsaccades.

## Results

A large field ‘on-off’ stimulation experiment was used to characterize the stimulus-locked (steady state evoked field, ‘SSVEF’) and broadband responses in visual cortex measured with MEG. The two measures are reported below, both prior to and after applying our new denoising algorithm Noisepool-PCA.

### Stimulus-locked and broadband signals measured with MEG

In each stimulus condition (both-, left-, and right-hemifield), the stimulus contrast reversed 12 times per second, so the stimulus-locked signal was measured at 12 Hz and harmonics. Because the largest component was at 12 Hz, we defined the stimulus-locked signal for a particular stimulus condition as the amplitude at 12 Hz, averaged over all 1-second epochs with that stimulus (typically ~180 epochs) computed for each of the 157 sensors in each subject (Fig 4; see Material and methods for details). The broadband signal was computed by averaging the log power across frequencies between 60 and 150 Hz, excluding multiples of the stimulus frequency (12 Hz), and then exponentiating the mean (Fig 4 inset; see Material and methods for details).

Both the stimulus-locked and broadband signals were largest in medial, posterior sensors, as expected from activations in visual cortex [46]. For the stimulus-locked signal, the both-hemifield condition tended to produce broadband signals in bilateral posterior sensors, whereas the single-hemifield conditions produced responses that were lateralized, with higher SNR contralateral to the stimulus. This pattern could be seen in an example subject and in the average across subjects (Fig 5). The lateralization of the stimulus-locked signal was less clear in the average across subjects due to imperfect alignment of the sensors showing the largest differential response to the left- and right- hemifield stimuli. In each of the 8 individual subjects and in each of the 3 conditions, the stimulus-locked response was evident, with the signal at least 10x above the noise (panel A in S5 Fig).

The spatial pattern of broadband signals was qualitatively similar to the spatial pattern of the stimulus-locked signal, with bilateral posterior responses in the both-hemifield condition, and lateralized responses in the single-hemifield conditions (Fig 5, individual example and group-averaged data). However, the broadband responses had much lower signal-to-noise than the stimulus-locked responses, and in many of the individual subjects, broadband was not evident in one or more conditions; for example, in the both-hemifield condition, there was a clear medial posterior broadband response for S1, S2, and S3, with signal at least 5x above noise, but not for S4 (panel B in S5 Fig). The broadband responses were less reliable for the left- and right-hemifield conditions than for the both-hemifield conditions.

The fact that broadband responses were evident in a few subjects in some conditions indicates that it is possible to measure broadband fields with MEG. However, if this signal cannot be measured reliably in many subjects and many conditions, then the practical value of measuring broadband with MEG is limited. This motivated us to ask whether denoising the MEG data could unmask broadband signals, making it more reliable across subjects and stimulus conditions.

### Denoising increases the broadband SNR by reducing variability

The MEG data were denoised using a new algorithm as described in detail in the Material and methods section. In brief, for each subject a subset of sensors that contained little to no stimulus-locked responses were defined as the noise pool. Once the noise pool was defined, the time series in each sensor and in each epoch was filtered to remove all signals not contributing to the broadband measurement. Global noise regressors were then derived by principal component analysis from the filtered time series in the noise pool in each 1-s epoch. The first 10 PCs were projected out of the data in each sensor, epoch by epoch. The remainder of the analysis was identical to that used in the non-denoised data set (Fig 2).

We first illustrate the effect of denoising with an example from a single sensor in one subject (Fig 6). This sensor showed a broadband response both prior to, and after, denoising. The benefit of denoising was not evident when comparing the mean power spectra before and after denoising (Fig 6A). Denoising did not reduce the variability in power across frequencies, nor did it increase the separation in the spectra for the contrast stimulus and the blank. Instead, the effects of denoising are better appreciated by examining the variability across epochs rather than across frequencies (Fig 6B). The biggest effect is that the broadband power estimates became less variable across epochs, both for the blank condition and the stimulus condition. This is indicated by the narrower distributions in the response amplitudes for the two conditions (Fig 6B, main panels) and for the difference between conditions (Fig 6B, insets). The standard deviation of the difference distributions decreased more than two-fold (from 0.91 to 0.34) as a result of denoising.

There are two other secondary patterns evident in these distributions. First, the mean broadband power of both the blank and stimulus condition decreased as a result of denoising (for the both-hemifield condition, 35.1 versus 26.1, prior to versus after denoising; for the blank, 28.2 versus 21.3). This was expected because projecting out signal reduces power. Second, the contrast between the two conditions (difference between the means) reduced: 6.9 prior to denoising versus 4.8 after denoising. The combination of these two effects was that the *percent difference* was little changed, with broadband power from the contrast-stimulus about 25% more than for the blank before and after denoising. Hence denoising did not increase the estimate of the percent signal change.

It is important to consider how these effects interact. Because the reduction in variability across epochs was the biggest effect of denoising (more than 2-fold), there was more than a doubling of SNR, computed as the mean across epochs divided by the variability of the difference distribution. In sum, the spectral plots show that the variability in power *across frequencies* was little affected by denoising (Fig 6A), whereas the distribution plots show that the variability in total broadband power *across epochs* was reduced considerably (Fig 6B).

We now consider the effect of denoising across sensors, subjects, and stimulus conditions. Projecting out noise PCs substantially increased the signal-to-noise ratio of the broadband measurement in visually responsive sensors. For example, in the both-hemifield condition for subject S1, the median SNR of the 10 most visually responsive sensors increased from 5 to 10 after denoising (Fig 7A, solid blue line), similar to the example sensor shown earlier (Fig 6B).

In contrast, the SNR of the 75 sensors in the noise pool was relatively unaffected by denoising (Fig 7A, dotted blue line). For these sensors, the SNR both prior to, and after denoising, was close to 0. This indicates that these sensors did not contain any appreciable stimulus-related broadband signal. This is important, because if the stimulus-related broadband response propagated to all sensors, then the noise pool would be contaminated by signal, and the algorithm might project out signal rather than reducing noise. The fact that the SNR increased in sensors in the back of the head, but was zero in the noise pool, is consistent with the interpretation that the algorithm removed global noise (as intended) rather than removing global signal.

Across the 8 subjects in the both-hemifield condition, taking the mean of the 10 most visually responsive sensors for each subject, the SNR increased about 3-fold (from 1.6 to 5.0), with a numerical increase in every subject (Fig 7B). Because the SNR stabilized in all subjects with 10 or fewer PCs projected out, in subsequent analyses, for simplicity we report the effects of denoising with exactly 10 PCs. A comparison of the SNR before denoising (0 PCs projected out) and after (10 PCs projected out) summarized across all subjects and the three stimulus conditions shows increases in SNR for every subject in all conditions (Fig 7C) (*p* < 0.001, *p* < 0.001, *p* < 0.01 for two-tailed paired t-tests, 0 v 10 PCs, for both-, left-, and right-hemifield conditions respectively).

In principle, the SNR increases could have arisen from increased signal, decreased noise, or both. To distinguish among these possibilities, we compared the signal level alone and the noise level alone before and after denoising. As in prior results, the signal was defined as the difference in broadband power between the contrast pattern and the blank (mean of data), and the noise was defined as the variability of this difference metric (standard deviation across bootstraps). For all three stimulus conditions in most subjects, the signal was largely unaffected by denoising, staying at a similar level or decreasing slightly, while the noise level went down substantially (Fig 8). These analyses indicate that the increase in SNR from denoising (Fig 7) was caused by a reduction in epoch-to-epoch variability of the broadband signal level, and not by an increase in the signal level, consistent with the results of the single example sensor (Fig 6). Expressed as a percentage increase over baseline, the broadband response to the both-hemifield stimulus after denoising was ~10.9±1.7% averaged across the top 10 sensors in each subject (mean ± sem across subject), and 12.6%±1.6% for the top 5 sensors. This contrasts with the much larger stimulus-locked response, which was a nearly 8-fold increase over baseline even prior to denoising (678%±226% increase over baseline for the top 5 sensors).

The effect of denoising the broadband signal was not uniform across the sensor array. In general, sensors where we expected visual activity (over the posterior, central part of the head) showed increased SNR following denoising. In the example subject S1 as well as the average across subjects, the denoised broadband response was observed in bilateral sensors for the both-hemifield condition, and with a contralateral bias (relative to the midline) in the two lateralized conditions (Fig 9). For the both-hemifield stimulus, broadband responses were evident in sensors over the posterior, middle of the head in most individual subjects (Fig 10).

For comparison, we conducted the above analyses on two synthetic data sets with known signal and noise (S4 Fig). For the synthetic dataset containing localized, stimulus-related broadband responses, the analysis reproduced several of the effects seen in the data. First, the denoising algorithm identified a noise pool of sensors that were not “visually responsive” (i.e. contained noise but no signal). Second, the algorithm increased the SNR in “visual” sensors (i.e., sensors with signal) but did not increase the SNR in the sensors with noise only. Third, the increase in SNR resulted from a decrease in noise rather than an increase in signal. Fourth, the SNR increased as the number of PCs projected out increased from 0 to 10, and then gradually decreased. This was expected because the global noise was a mixture of 10 basis functions. For the synthetic dataset that did not contain localized, stimulus-related broadband responses, applying the algorithm did not add artefactual broadband responses.

The above analyses were conducted with a few specific choices made with respect to how we denoised: the number of PCs projected out (10 PCs), the size of the noise pool (75 sensors), and the number of 1-s epochs denoised at a time (one at a time). In separate analyses, we swept out a wide range of these parameters (S1 and S2 Figs). These results showed that the most SNR was gained for (1) intermediate numbers of sensors in the noise pool (50–90) and intermediate number of PCs removed (10–50) (S1 Fig), and (2) a small number of epochs denoised at a time (S2 Fig).

### Control analyses for Noisepool-PCA algorithm

To validate the assumptions in our denoising algorithm, we ran three control analyses. In one control analysis, we concatenated all epochs to derive noise regressors from the whole experimental time series (Fig 11, 2^nd^ bar, compared to using the default of 1-s epochs to derive noise regressors– 1^st^ bar). The elevation in broadband SNR was significantly less when we concatenated all epochs (*p* < 0.01, *p* < 0.01, *p* < 0.05 for both-, left-, and right-hemifield stimulus conditions, respectively). In the second control analysis, the noise pool included all sensors rather than only those sensors that were not visually responsive. Here, the noise regressors included some signal as well as noise, and hence should be of less benefit. This expectation was confirmed, in that there was no increase in SNR when the algorithm was run with the omission of the noise-pool-selection step (Fig 11, 3^rd^ bar, *p* < 0.01 for all the three stimulus conditions). In a 3^rd^ control analysis, we phase-scrambled each of the epoch-by-epoch noise time series. The phase-scrambled regressors were temporally uncorrelated with the actual time series in the noise. As a result, we found no change in SNR levels (Fig 11, fourth bar, *p* < 0.001, *p* < 0.001, *p* < 0.05 for both-, left-, right-hemifield stimulus conditions, respectively).

### Other denoising algorithms

To assess how other existing denoising algorithms affect our measurement of broadband power, and how they interact with our new denoising algorithm, we ran two different denoising algorithms, either alone or in combination with Noisepool-PCA. The two algorithms we tested were CALM, or continuously adjusted least-square method [38] and TSPCA, or time-shift principal component analysis [39]. Both of these make use of reference MEG sensors which face away from the head and measure environmental rather than physiological fields. By design, these algorithms project out time series from the subspace spanned by the reference sensors, thereby reducing environmental noise, but not physiological noise. Applying either one of these two algorithms alone to the 8 data sets reported above increased the broadband SNR, evident in the group-averaged sensor plots (Fig 12A, columns 3–4 versus column 2), and the increased SNR in the 10 most responsive sensors (Fig 12B, 2^nd^ and 3^rd^ bar versus 1^st^ bar in each plot).

In planned comparisons, we evaluated the SNR increase of each algorithm or combination of algorithms to the increase from Noisepool-PCA alone. The increase from each of the two environmental algorithms alone or from no denoising was significantly less than that from our new Noisepool-PCA algorithm (Fig 12A, column 5 versus columns 2–4; Fig 12B, 4^th^ bar versus 1^st^-3^rd^). Applying two algorithms in sequence, first either CALM or TSPCA, followed by Noisepool-PCA, also resulted in a large increase in broadband SNR (Fig 12A, columns 6 and 7). For all three stimulus conditions, the combination of Noisepool-PCA and CALM resulted in the numerically largest gain in SNR, which was significantly larger than Noisepool-PCA alone for the both- and left-hemifield condition (*p* < 0.05, Fig 12B, 5^th^ versus 4^th^ bars), with no trend toward a larger gain for the right-hemifield condition (*p* = 0.1343). This suggests that the Noisepool-PCA algorithm and an environmental algorithm captured some independent noise.

### Effect of denoising on stimulus-locked SNR

In a separate analysis, we ran the Noisepool-PCA algorithm to evaluate its effect on the stimulus-locked signal. The methods were identical to those used to denoise the broadband signal except for the omission of one step, the step in which we filtered the time series to remove temporal components that do not contribute to the broadband signal. Denoising modestly increased the stimulus-locked SNR for all stimulus conditions for most subjects (S7 Fig, top). The SNR increased numerically in all subjects (n = 8) and in all stimulus conditions, although the percentage increases were smaller than those for denoising the broadband signal, ~20% increase compared to two-fold. This smaller benefit would be expected if the stimulus-locked signal, being limited to a single temporal frequency, were less contaminated by noise: noise at any temporal frequency could affect the broadband measure, whereas only noise at the stimulus-locked frequency would affect the stimulus-locked measure. As in the case of denoising the broadband signals, the main contribution to the increase in SNR for the stimulus-locked signal was a decrease in variability across epochs (S7 Fig, bottom), rather than an increase in the signal level (S7 Fig, middle).

### Broadband fields measured with Elekta 360 Neuromag

To test whether the findings reported above generalize to other instruments and experimental environments, we conducted the same experiment using a different type of MEG system, an Elekta 360 Neuromag at CiNet. The CiNet system contains paired planar gradiometers, in contrast to the axial gradiometers used in the Yokogawa MEG at NYU, and the scanner is situated in a different physical environment, with potentially very different sources of environmental noise. The preprocessing pipeline at this imaging center often includes a denoising step based on temporally extended signal source separation (tSSS) [40, 41]. This additional experiment gave us the opportunity to ask several questions: (1) Are broadband fields observed with a different MEG sensor type and different physical environment? (2) Does the tSSS algorithm increase the broadband SNR? (3) Does our new Noisepool-PCA algorithm increase the SNR of data that have already been denoised with the tSSS algorithm?

The identical experiments were conducted with 4 new subjects. As expected, all three stimulus types led to a large stimulus-locked response in the posterior sensors, with a peak SNR of more than 10 in the group averaged data (Fig 13A, column 1). A modest, spatially specific broadband signal was measured from the data before denoising for each stimulus type (Fig 13A, column 2), with a peak SNR of 1–2 in the group averaged data for all three conditions. Unlike the NYU data, in the CiNet data the Noisepool-PCA algorithm on the raw data did not generally result in an increase in the broadband SNR (group data, Fig 13A, columns 2 and 3; individual subjects, Fig 13B, left side of each subplot). However, when the raw data were preprocessed with the tSSS algorithm (Fig 13A, column 4), application of Noisepool-PCA increased the SNR in all 3 stimulus conditions for 3 out of 4 subjects, and in 2 out of 3 stimulus conditions for the 4th subject. Together, the Noisepool-PCA algorithm increased the SNR by 2–3 fold, similar to the NYU data (both-hemifield: 3.0 to 5.4; left-hemifield: 0.70 to 2.6; right-hemifield: 2.1 to 4.3; means across 4 subjects S9-S13, top 10 sensors each, for the tSSS data and the Noisepool-PCA denoised tSSS data). Just as with the NYU MEG data set, the combination of an algorithm tailored to find environmental noise (tSSS) and our algorithm produced the most robust results, indicating that Noisepool-PCA and the environmental denoising algorithm removed at least some independent sources of noise.

### Saccadic eye movements during MEG experiments

Saccadic eye movements are known to have a large influence on MEG and EEG measurements. This influence can be especially pernicious when measuring high frequency broadband signals, because the spike field (MEG) or spike potential (EEG) arising from extraocular muscle contraction can be spectrally broadband and can co-vary with task design; hence, it can easily be confused with broadband signals arising from brain activity [22, 24]. For visual experiments, the spike potential in EEG is especially problematic because it tends to affect sensors which are also visually sensitive (posterior middle). In contrast, the MEG spike field is lateral, potentially influencing temporal and frontal sensors, with little to no effect on posterior sensors [21]. Hence spike field artifacts are unlikely to contaminate our visually elicited broadband signals, which are most clearly evident in the central posterior sensors.

Nonetheless, for a subset of subjects (S6-S8), we measured eye movements during the MEG experiments and quantified the frequency of microsaccades, and the distribution of microsaccade direction, for each stimulus condition. Each of these 3 subjects showed broadband responses in their denoised data (Fig 10). All three subjects showed a higher rate of horizontal than vertical microsaccades in every stimulus condition (Fig 14A), consistent with prior observations [47], but there was no systematic pattern in saccade frequency as a function of stimulus condition; for example, the stimulus condition with the most and with the fewest microsaccades differed across the 3 subjects. Moreover, the subject with the highest broadband SNR among these 3 (S6) had the lowest rate of microsaccades (~0.5 microsaccades / second). To test more directly whether microsaccades contributed to the measured broadband fields, we re-analyzed the data from these 3 subjects in two ways, either limited to only those epochs with microsaccades or only those epochs without microsaccades (Fig 14B). The broadband responses were evident in each subject in the epochs without microsaccades, indicating that this response is not entirely an artifact of microsaccades.

## Discussion

We separated the MEG signal into two components, one time-locked and one asynchronous with the stimulus. The stimulus-locked component was clearly visible in all subjects with minimal preprocessing. The asynchronous signal, spanning 60–150 Hz, was visible with little preprocessing in some subjects and the mean across subjects. However, the SNR was low compared to the stimulus-locked component. With our denoising algorithm, SNR more than doubled, resulting in reliable, spatially specific broadband signals in all individuals. We showed in a subset of subjects that the broadband signals could not be explained by systematic biases in the pattern of fixational eye movements, supporting the interpretation that the broadband fields arise from neural activity rather than artifacts associated with eye movements.

These results are *qualitatively* consistent with intracranial measurements [27]. However, it has proven difficult to measure extracranial broadband signals arising from neural activity. Below, we discuss the significance of broadband responses, challenges in measuring them extracranially, and the generalizability of our denoising algorithm.

### Significance of broadband responses

A century ago Berger and others described oscillations in surface EEG between 10 and 25 Hz [3, 48]. More recently, using intracranial recordings, Crone and colleagues [49] reported power increases in higher frequencies (75–100 Hz) associated with motor movements. Subsequently, this high frequency response has been observed throughout cortex (S8 Fig), and has been interpreted as a broadband (not oscillatory) signal, thought to reflect increased neuronal activity, rather than increased synchrony [4, 5]. Two studies proposed computational models linking the rate of spike arrivals to broadband power in the field potential [8, 11]. Consistent with these models, studies with simultaneous measurement of spiking and LFP have shown that broadband signals correlate with single [12] and multiunit spike rates [9, 10]. Under some conditions, broadband is also correlated with the BOLD signal [14, 18, 27]. BOLD signals, however, are influenced by processes other than spiking [50–52]; hence quantifying broadband responses from the same experimental paradigm studied with fMRI can help disentangle the relative contribution to the observed BOLD response from spiking versus other, non-spiking neural activity. Being able to reliably measure the broadband signal extracranially offers the opportunity to noninvasively measure neuronal spiking activity at a sub-second scale, complementing fMRI and oscillatory and time-locked (evoked) signals commonly measured with MEG and EEG.

### Prior measures of extracranial broadband and gamma band responses

#### Broadband vs. narrowband gamma

Several groups have distinguished broadband power increases from narrowband gamma oscillations [7, 10]. Gamma oscillations are observed in visual cortex for some stimuli (e.g., high contrast gratings) [53, 54], with a peak frequency between 30 and 100 Hz and bandwidth of 10–20 Hz. The broadband response occurs in many brain areas for many types of stimuli, spanning at least 50–150 Hz, and can extend to lower and higher frequencies [5, 27]. Robust gamma oscillations have been measured extracranially for grating stimuli [29, 30]. Because this signal originates from synchronized neural activity [55], it propagates efficiently over distances [56], similar to the stimulus-locked signal we discussed above. These oscillations differ from the broadband fields reported here, which span a wider frequency range, have lower amplitude, and likely reflect asynchronous neural activity.

#### Multiple gamma peaks

Some extracranial studies have reported multiple distinct signals within the gamma band. For example, Wyart and Tallon-Baudry [57] and Vidal *et al*. [31] measured MEG responses to gratings and bars, respectively. They reported power increases in two bands, from 45–65 Hz and 75–120 Hz. Both components were interpreted as oscillations arising from synchronous neural activity, and are likely different from the broadband signals reported here.

#### Group averaged broadband

Two MEG studies reported increases in high gamma power (60–140 Hz) during recall of visual stimuli [58, 59]. These studies showed averages across subjects (22 or 24), so that it is not known whether there were reliable responses in individuals. Moreover, these power increases were interpreted as a change in neural synchrony within this frequency range, whereas we interpret our results as arising from a change in the level of neuronal activity.

#### Motor cortex

High frequency signals (~65–100 Hz) have been shown from motor cortex measured extracranially [32, 33]. This signal was most evident in group-averaged data and some but not all individuals, and within a relatively narrow band (~20–30 Hz wide). Ball *et al*. [32] noted that better methods for measuring high frequency broadband extracranially would help resolve whether individual differences were due to measurement limitations or the lack of high frequency brain signals in some subjects. Cheyne *et al*. [60] measured high gamma (65–80 Hz) with MEG over motor cortex in individual subjects, and speculated that these signals reflect cortico-basal ganglia loops, as the basal ganglia is known to produce narrowband oscillations peaked at 70–80 Hz.

#### Source localized broadband

Two studies measured extracranial broadband signals indirectly, projecting the sensor activity to cortical sources. One study used independent component analysis (ICA) of EEG signals to identify high-frequency broadband responses during mental imagery and emotion tasks [61]. The other study analyzed MEG signals during two-person communication tasks and observed broadband spectral elevations in a source-localized, group analysis (n = 48) [62]. Our study differs from these in that we observe broadband responses in individual subjects and individual sensors, and, unlike the case of ICA, we do not manually select components of interest (see also below, ‘Noisepool-PCA and other denoising algorithms’).

### Challenges in measuring extracranial broadband responses

#### Extracranial broadband signal strength is low

Although having high SNR after denoising, the MEG broadband signal was nonetheless small relative to baseline—about a 13% increase. Using nearly the identical stimulus, the broadband signal measured by ECoG was ~15 times larger (a ~190% increase) [27]. The discrepancy was much smaller for the stimulus-locked signal (almost 8-fold increase with MEG vs. 21-fold with ECoG). Why are MEG broadband signals small? First, MEG sensors pool over a large area, so baseline power reflects activity from a large fraction of the brain, whereas visually driven broadband responses likely come from confined regions [63]. In contrast, both baseline and visual responses in ECoG electrodes arise from the same cortical patch. Second, the amplitude depends not only on pooling area, but also phase coherence. If broadband signals arise from incoherent neural activity, and stimulus-locked signals from coherent (synchronous) activity, then the former will grow with the square-root of the number of sources, and the latter with the number of sources. Since MEG pools over much larger populations than ECoG, the ratio of incoherent signal strength (broadband) to coherent (stimulus-locked) will be much lower. This logic is supported by modeling [56] and empirical studies with intra- and extracranial measures, which found that the most coherent intracranial signals were best transmitted outside the head [34, 64].

#### Extracranial measurements contain multiple noise sources

Because extracranial broadband power is low, noise is a major impediment. In addition to neural noise [65], fixational eye movements [24], head muscle contraction [66], and environmental perturbations [25] produce noise measured by MEG and EEG sensors. Many of these noise sources are spectrally broad and hence particularly problematic when investigating neural broadband signals.

Although spike fields generated from eye movements can be mistaken for broadband neural activity [22], it is unlikely that our spatially-specific broadband measures were substantially contaminated by eye movement artifacts. This was confirmed by analyses of eye movement data, and the fact that middle posterior sensors where we observed broadband are not usually associated with MEG spike field artifacts [21]. A second eye movement confound, the electromagnetic fields arising from movement of the retina-to-cornea dipole, causes low frequency artifacts (4–20 Hz; [44]) and therefore is unlikely to have affected our broadband measures (60–150 Hz).

Head muscles can also cause spectrally broadband contaminants [66], as can external noise sources, e.g., nearby electrical equipment. However, these noise sources are unlikely to be confined to occipital sensors and to co-vary with stimulus condition, and hence do not explain our broadband observations. Moreover, it is likely that these noise sources, if present, were included in our noise pool, and hence Noisepool-PCA would have reduced their effects.

The amplitude of the broadband signal we measure differed between participants. This could arise from differences in the level of neuronal broadband, as found with ECoG measures [67], from differences in physiological noise sources, or differences in cortical head geometry and MEG sensitivity to visual cortex. Due to the relatively small number of participants in the study, we did not try to infer which of these factors contributes most to individual differences in measured MEG broadband.

### Noisepool-PCA and other denoising algorithms

The goal of the Noisepool-PCA algorithm is to estimate, and then project out, noise in order to get a more reliable measure of signal. This approach is likely to yield unstable results when the noise level is too high, for example in the case of faulty sensors or brief periods with unusually large external disturbances. Therefore, as a preprocessing step prior to the application of the algorithm, we first identified and rejected sensors and epochs with very high noise levels. This procedure followed simple rules based on the overall variance in the MEG time series, described in the Material and methods. There are other algorithms for identification of bad sensors or epochs such as the ‘Autoreject’ algorithm [68] and any such algorithm could substitute for our preprocessing step.

Noisepool-PCA defines noise regressors by applying PCA on a subset of sensors. In principle, the procedure can capture any noise source contributing to the noise pool, including environmental, oculomotor, muscular, and neural. This differs from algorithms designed to remove environmental noise. Hence Noisepool-PCA is complementary to these methods. After the initial step of rejecting bad sensors and bad epochs, we found that the most effective analysis was either Noisepool-PCA alone, or Noisepool-PCA following an environmental denoising algorithm such as CALM [38], TSPCA [39], or TSSS [40, 41]. For general usage, it therefore seems prudent to first reject bad sensors and epochs, and then to use an environmental denoising algorithm followed by our new method.

Our algorithm has much in common with other denoising approaches that rely on spatial decomposition, including ICA denoising [43] and SSP (Signal Space Projections) [69], with some important differences. First, PCA, unlike ICA and SSP, ranks components by variance explained. Second, Noisepool-PCA explicitly identifies noise sensors. These features enable the algorithm to be fully automated, making it easy to denoise at the time scale of individual events (e.g., >1,000 one-second epochs). If the spatial pattern of the PCs varies over time, then deriving the components independently within short epochs is more effective (Fig 11; S2 Fig).

To use Noisepool-PCA for other experimental designs, analyses, or scanners, one would need to change some input parameters. In addition to the experimental design matrix and data, required inputs include the experiment-specific functions to summarize the MEG responses. In our experiments, one function computed the stimulus-locked signal and was used to define the noise pool. For most of our analyses, a second function computed the broadband power to evaluate the results. In principle, one could use a single function to define the noise pool and evaluate the data (as we did for denoising the stimulus-locked signal). For other experiments, one might use a function that computes the amplitude or latency of an evoked response, or the power in a limited temporal frequency band, or any measure relevant to the experiment. Alternatively, one could run a separate localizer experiment to identify a pool of potential sensors of interest and a pool of noise sensors, and then manually enter the list of noise sensors to denoise the main experiment. Unlike ICA or SSP denoising, our algorithm will not work on resting state data for which no task design is available. There are several other optional inputs, such as the method to identify the noise pool, the accuracy metric (SNR/R^2^). Here, we used the defaults for all optional inputs. The algorithm might not be suitable for experimental paradigms in which all sensors carry responses that are related to the structure of the experiment. However, it is likely useful for many sensory experiments or other paradigms that strongly drive responses in localized regions of the brain.

### Conclusion

Stimulus-driven broadband brain responses can be quantitatively characterized in individual subjects using a non-invasive method. Because we obtain high SNR measures from short experiments, the broadband signal can be used to address a wide range of scientific questions. Access to this signal opens a window for neuroscientists to study signals associated with neuronal spike rates non-invasively at a high temporal resolution in the living human brain.

## Supporting information

S1 Fig The effect of number of sensors in the noise pool and number of principle components (PCs) removed on denoising broadband SNR for the top 10 sensors with the highest SNR across the three stimulus conditions, either before or after denoising. Colors represent difference in SNR before and after denoising. Left, middle and right panel correspond to both-, left-, right-hemifield stimulus. All three conditions show highest increase in broadband SNR when there are 50–80 sensors in the noise pool and 10–50 PCs removed from the data. Control datasets are made with *nppDenoiseNPCvsNoisepool*.*m*. Figure made with function *nppMakeFigureS1*.*m*.
(TIFF)

S2 Fig The effect of epoch length on denoising broadband SNR for the top 10 sensors with highest SNR. Left, middle and right panel correspond to both-, left-, right-hemifield stimulus. Noise pool was defined as 75 sensors and fixed across epoch lengths and number of PCs removed. (A) Difference in broadband SNR before and after denoising when varying the length of one epoch. All epochs depend on subject number but ranged between a total time of ~12–15 minutes. All three conditions show highest increase in broadband SNR when the epoch length is 3 seconds or lower. (B) Difference in broadband SNR before and after denoising when varying the numbers of epochs denoised at the same time and varying the number of principle components (PCs) removed from the data. All conditions show a max increase in SNR between 1–6 epochs, removing 10–70 for the both-hemifield condition and 30–60 for left- or right-hemifield conditions. Control datasets are made with *nppDenoiseVaryEpochLength*.*m*. Figure made with function *nppMakeFigureS2*.*m*.
(TIFF)

S3 Fig Summary statistics of PCs from the noise pool from one subject. (A) Percentage of variance explained by PCs in time series for each epoch (each line is one epoch), with inset zooming in on the first 10 PCs. Dashed line represents the mean across epochs. (B) Mean spectrum of noise time series across epochs, for the first 10 PCs (black lines) and the average (red line). (C) Noise time series of one epoch of PC 2 (black line) and the envelope of the noise time series (red line). (D) Amplitude spectrum of the envelope shown in C. (E) Mean amplitude spectra of envelopes across epochs and first ten 10 PCs. Grid lines mark 12 Hz frequency and harmonics up to 72 Hz. Figure made with function *nppMakeFigureS3*.*m*.
(TIFF)

S4 Fig Effect of Noisepool-PCA on broadband responses in synthetic data set with (A-F) and without stimulus-related broadband (G-L). (A) Spectral power of both-hemifield (blue), and blank screen (gray) conditions from one sensor in synthetic data set (black dot in inset). (B) Same data as panel A, but zoomed to higher frequencies (60–150 Hz) to emphasize the broadband component (orange box in A). Prior to denoising, there is little difference between the broadband power in the two conditions, stimulus and blank. (C) Same as panel B, but after running Noisepool-PCA on the synthetic dataset. As with actual MEG data, we removed the harmonics of the stimulus-locked component (12 Hz). After denoising, the stimulus condition has more broadband power than the blank condition. The overall power declines for both stimulus and blank conditions (see Fig 6 for similar results on MEG data). (D) SNR as a function of number of principle components (PCs) projected out. The SNR rises until 10 PCs are projected out, and then slowly declines, similar to MEG data (Fig 7). This is expected because there is noise in each sensor mixed from 10 basis functions. The inset shows the noise pool (yellow) and sensors of interest in gray. (E) Topographic map of broadband SNR before denoising. (F) Topographic map of broadband SNR after denoising (projecting out 10 PCs). Both panel E and F use the color bar on the right. (G-L) Plotting conventions as panels A-F but for synthetic dataset without stimulus-related broadband signals (i.e. setting the broadband response amplitude to 0). Our algorithm chooses the same sensors in the noise pool, but this does not result in an increased broadband SNR after denoising. This result indicates that we are not artificially injecting broadband responses. Synthetic datasets are made with *nppMakeSyntheticDataSet*.*m*. Figure made with function *nppMakeFigureS4*.*m*.
(TIFF)

S5 Fig Topographic maps of stimulus-locked and broadband SNR in individual subjects before denoising. (A) Stimulus-locked SNR for the both-, left-, right-, and left minus right-hemifield stimulus, without denoising. Rows 1–4 use the SL SNR color bar. (B) Broadband SNR before denoising. Rows 1–3 use the BB SNR color bar, and the 4^th^ row uses the BB L-R SNR color bar. Made with function *nppMakeFigureS5*.*m*.
(TIFF)

S6 Fig Topographic maps of broadband SNR in individual subjects after denoising. Head plots show the stimulus-locked SNR for the both-, left-, right- and left minus right-hemifield stimulus, after denoising. Rows 1–3 use the BB SNR color bar, and the 4^th^ row uses the BB L-R SNR color bar. Made with function *nppMakeFigureS6*.*m*.
(TIFF)

S7 Fig Denoising the stimulus-locked signal. The Noisepool-PCA algorithm results in a modest increase in SNR for most subjects in all three stimulus conditions (top row). This benefit is largely due to the fact that the noise level goes down from denoising (bottom row) rather than the signal increasing (middle row). Plotting conventions as in Figs 7C and 8. Made with function *nppMakeFigureS7*.*m*.
(TIFF)

S8 Fig Broadband signals around the brain. ECoG studies have measured broadband power elevations associated with perception, movement, language, and cognition [6, 70–72] Examples of broadband field potentials from single ECoG electrodes in motor cortex (A), ventral temporal cortex (B), and primary visual cortex (C). The power increases relative to baseline span at least 50 to 200 Hz. Adapted from (A) [73]; (B) [6]; (C) [74].
(TIFF)
