# Supplementary figures and images for: A non-invasive, quantitative study of broadband spectral responses in human visual cortex
Source: PLoS One. 2018 Mar 12;13(3):e0193107. doi: 10.1371/journal.pone.0193107 (PMC5846788; doi:10.1371/journal.pone.0193107)

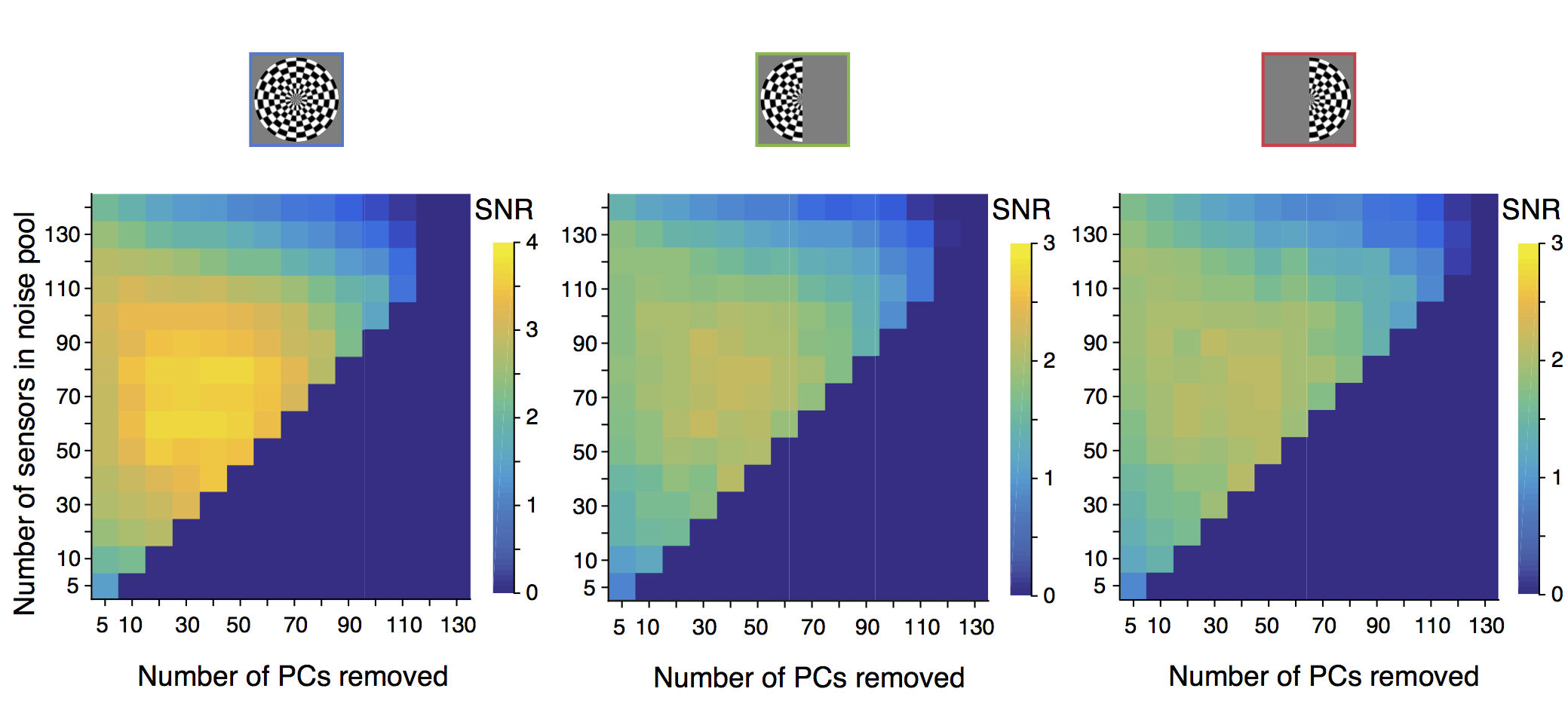

Supplement: S1 Fig — Colors represent difference in SNR before and after denoising. Left, middle and right panel correspond to both-, left-, right-hemifield stimulus. All three conditions show highest increase in broadband SNR when there are 50–80 sensors in the noise pool and 10–50 PCs removed from the data. Control datasets are made with nppDenoiseNPCvsNoisepool.m. Figure made with function nppMakeFigureS1.m. (TIFF) [file pone.0193107.s001.tiff]

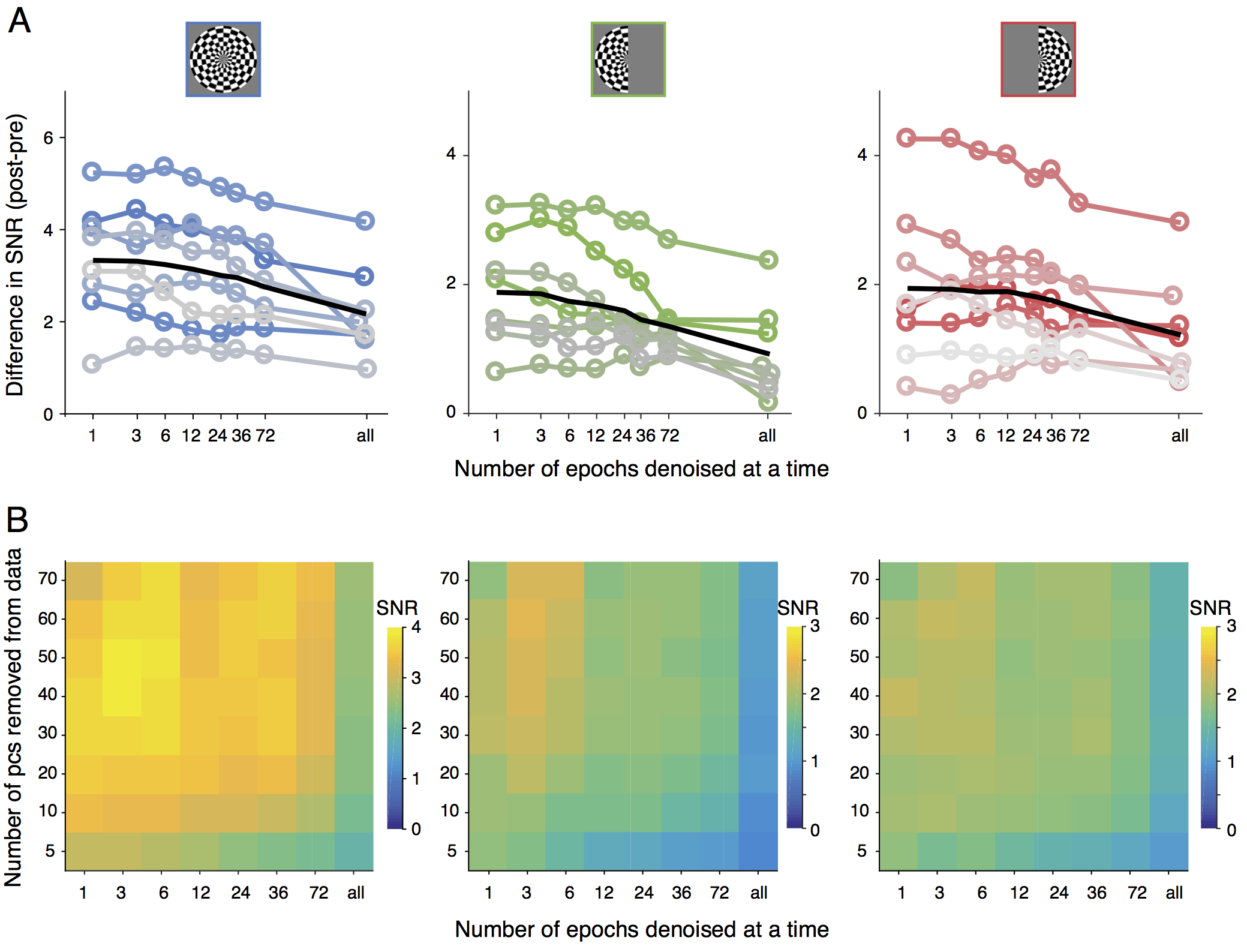

Supplement: S2 Fig — Left, middle and right panel correspond to both-, left-, right-hemifield stimulus. Noise pool was defined as 75 sensors and fixed across epoch lengths and number of PCs removed. (A) Difference in broadband SNR before and after denoising when varying the length of one epoch. All epochs depend on subject number but ranged between a total time of ~12–15 minutes. All three conditions show highest increase in broadband SNR when the epoch length is 3 seconds or lower. (B) Difference in broadband SNR before and after denoising when varying the numbers of epochs denoised at the same time and varying the number of principle components (PCs) removed from the data. All conditions show a max increase in SNR between 1–6 epochs, removing 10–70 for the both-hemifield condition and 30–60 for left- or right-hemifield conditions. Control datasets are made with nppDenoiseVaryEpochLength.m. Figure made with function nppMakeFigureS2.m. (TIFF) [file pone.0193107.s002.tiff]

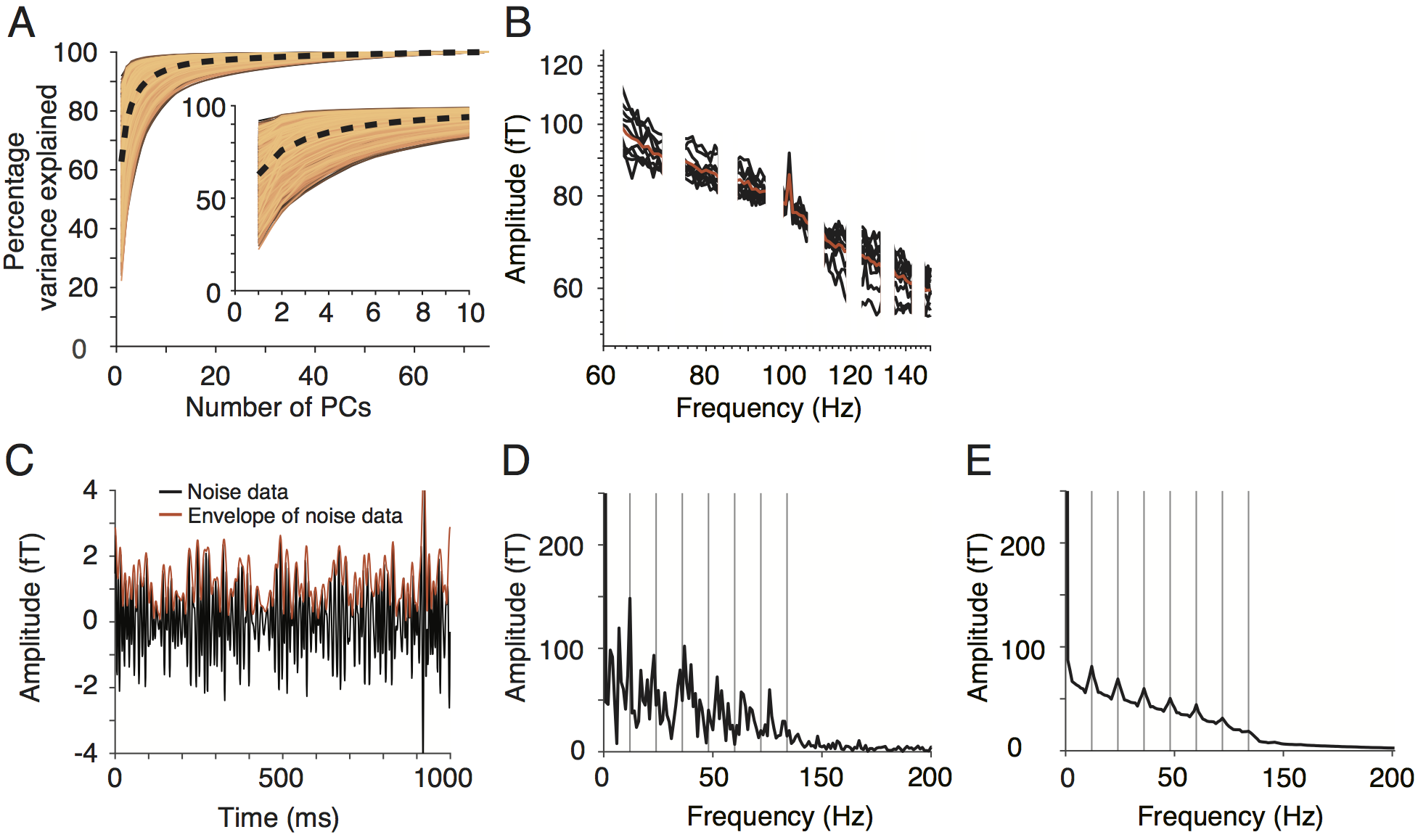

Supplement: S3 Fig — (A) Percentage of variance explained by PCs in time series for each epoch (each line is one epoch), with inset zooming in on the first 10 PCs. Dashed line represents the mean across epochs. (B) Mean spectrum of noise time series across epochs, for the first 10 PCs (black lines) and the average (red line). (C) Noise time series of one epoch of PC 2 (black line) and the envelope of the noise time series (red line). (D) Amplitude spectrum of the envelope shown in C. (E) Mean amplitude spectra of envelopes across epochs and first ten 10 PCs. Grid lines mark 12 Hz frequency and harmonics up to 72 Hz. Figure made with function nppMakeFigureS3.m. (TIFF) [file pone.0193107.s003.tiff]

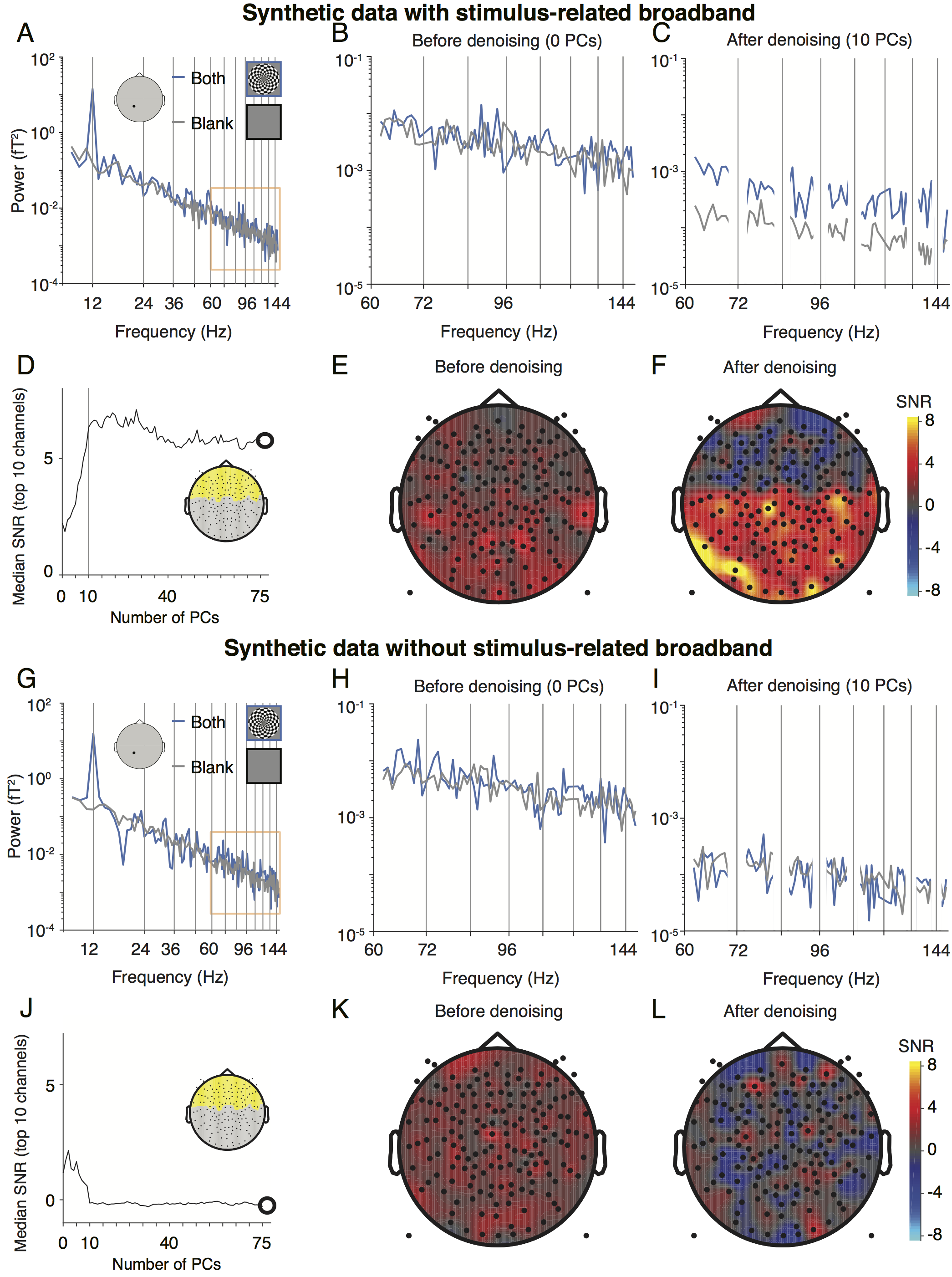

Supplement: S4 Fig — (A) Spectral power of both-hemifield (blue), and blank screen (gray) conditions from one sensor in synthetic data set (black dot in inset). (B) Same data as panel A, but zoomed to higher frequencies (60–150 Hz) to emphasize the broadband component (orange box in A). Prior to denoising, there is little difference between the broadband power in the two conditions, stimulus and blank. (C) Same as panel B, but after running Noisepool-PCA on the synthetic dataset. As with actual MEG data, we removed the harmonics of the stimulus-locked component (12 Hz). After denoising, the stimulus condition has more broadband power than the blank condition. The overall power declines for both stimulus and blank conditions (see Fig 6 for similar results on MEG data). (D) SNR as a function of number of principle components (PCs) projected out. The SNR rises until 10 PCs are projected out, and then slowly declines, similar to MEG data (Fig 7). This is expected because there is noise in each sensor mixed from 10 basis functions. The inset shows the noise pool (yellow) and sensors of interest in gray. (E) Topographic map of broadband SNR before denoising. (F) Topographic map of broadband SNR after denoising (projecting out 10 PCs). Both panel E and F use the color bar on the right. (G-L) Plotting conventions as panels A-F but for synthetic dataset without stimulus-related broadband signals (i.e. setting the broadband response amplitude to 0). Our algorithm chooses the same sensors in the noise pool, but this does not result in an increased broadband SNR after denoising. This result indicates that we are not artificially injecting broadband responses. Synthetic datasets are made with nppMakeSyntheticDataSet.m. Figure made with function nppMakeFigureS4.m. (TIFF) [file pone.0193107.s004.tiff]

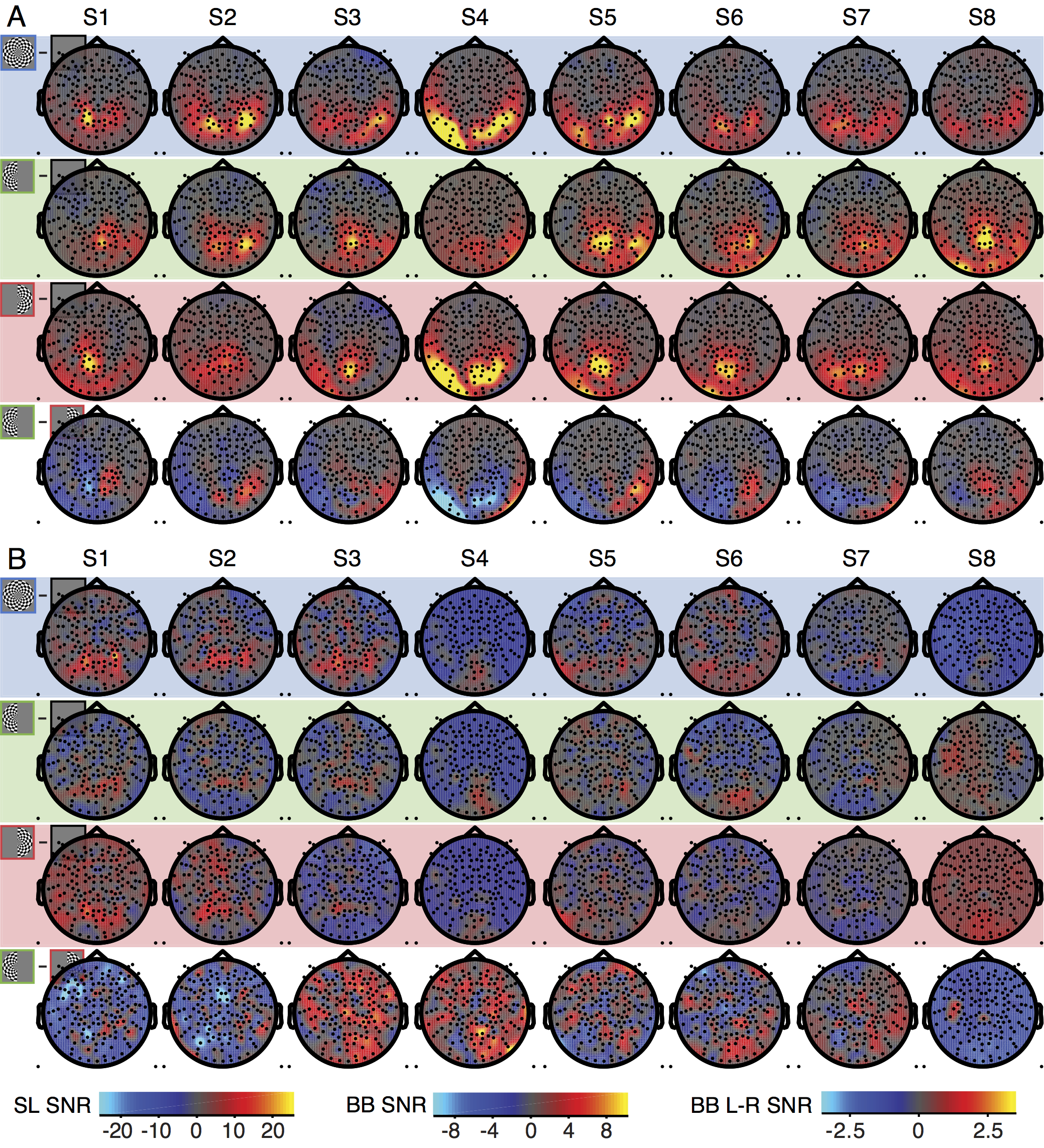

Supplement: S5 Fig — (A) Stimulus-locked SNR for the both-, left-, right-, and left minus right-hemifield stimulus, without denoising. Rows 1–4 use the SL SNR color bar. (B) Broadband SNR before denoising. Rows 1–3 use the BB SNR color bar, and the 4th row uses the BB L-R SNR color bar. Made with function nppMakeFigureS5.m. (TIFF) [file pone.0193107.s005.tiff]

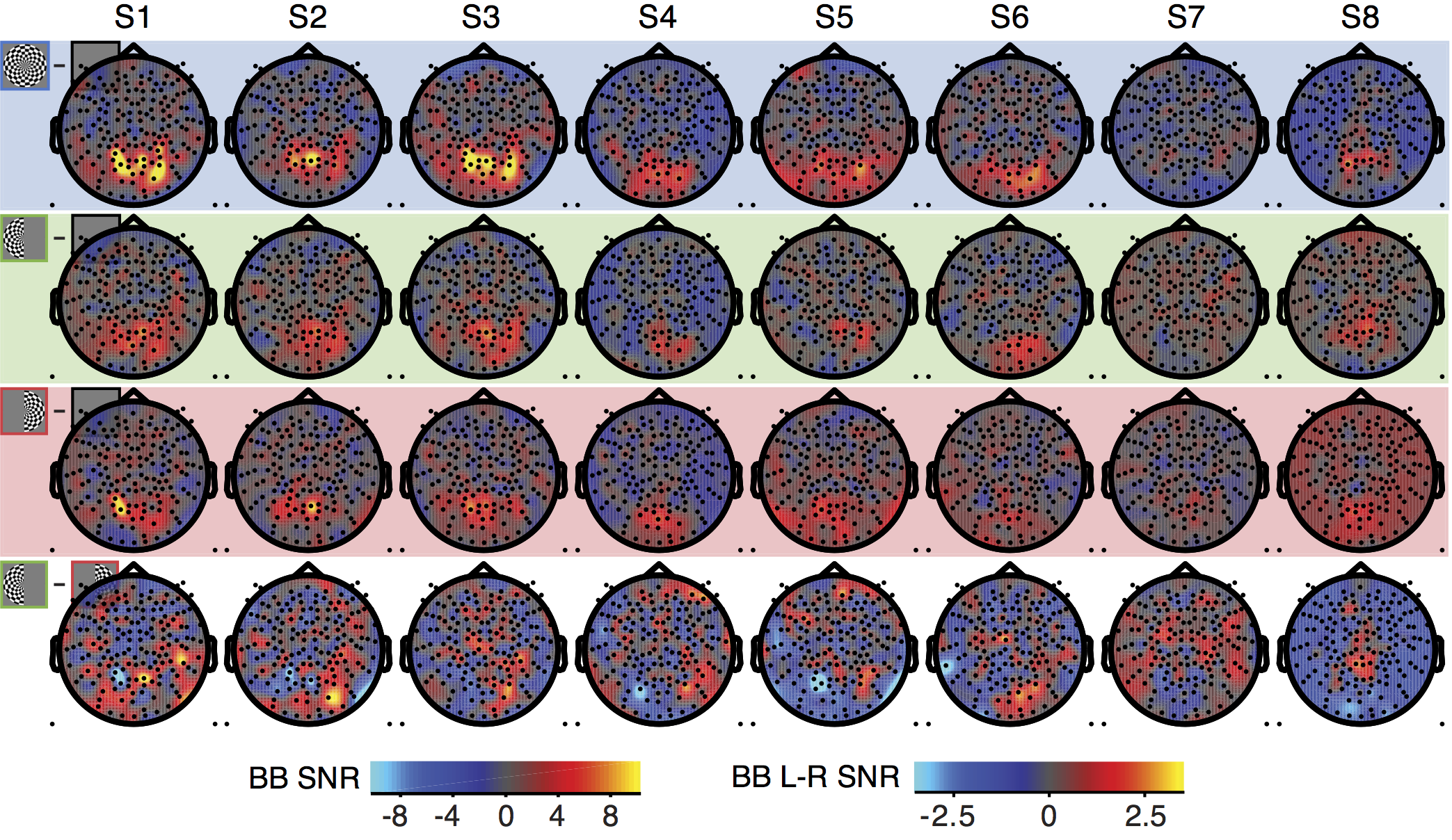

Supplement: S6 Fig — Head plots show the stimulus-locked SNR for the both-, left-, right- and left minus right-hemifield stimulus, after denoising. Rows 1–3 use the BB SNR color bar, and the 4th row uses the BB L-R SNR color bar. Made with function nppMakeFigureS6.m. (TIFF) [file pone.0193107.s006.tiff]

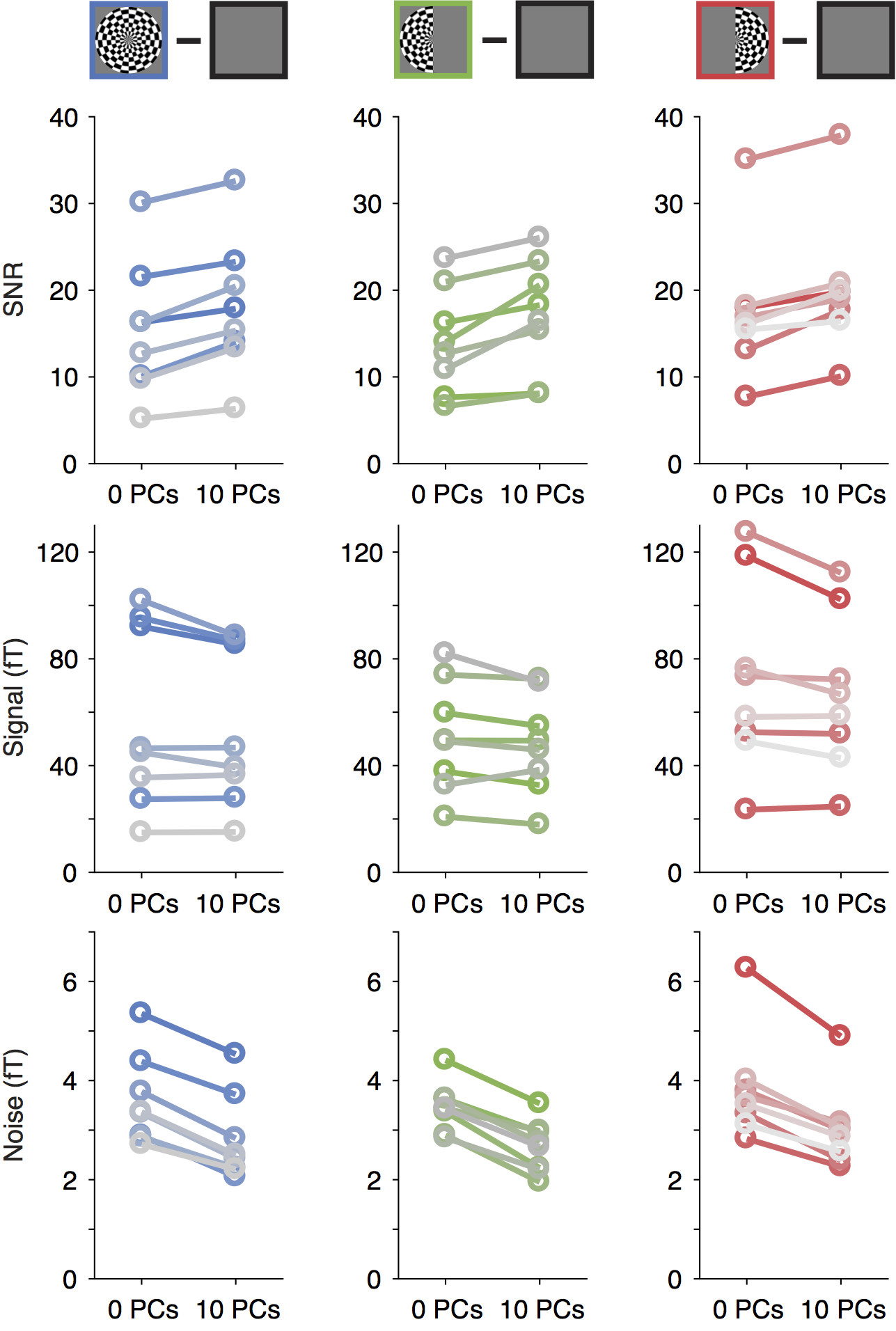

Supplement: S7 Fig — The Noisepool-PCA algorithm results in a modest increase in SNR for most subjects in all three stimulus conditions (top row). This benefit is largely due to the fact that the noise level goes down from denoising (bottom row) rather than the signal increasing (middle row). Plotting conventions as in Figs 7C and 8. Made with function nppMakeFigureS7.m. (TIFF) [file pone.0193107.s007.tiff]

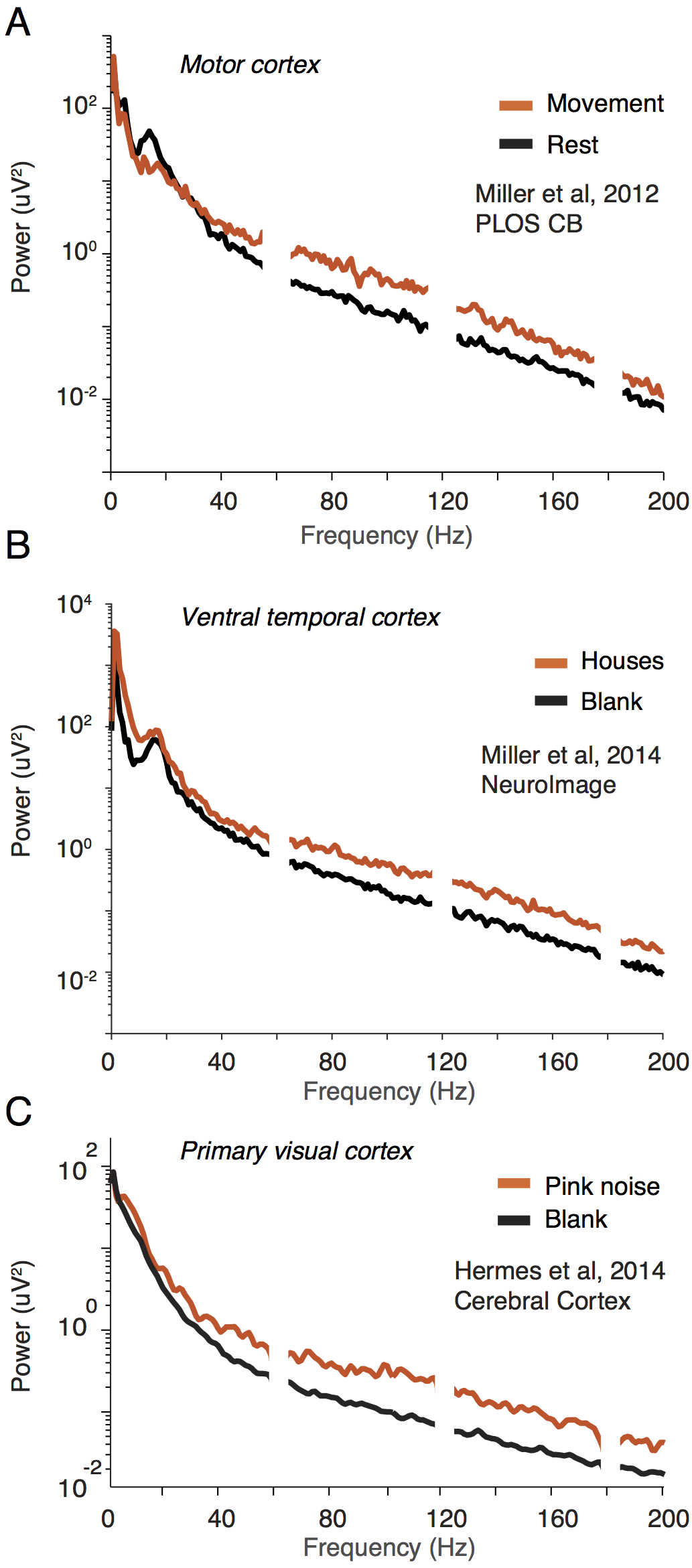

Supplement: S8 Fig — ECoG studies have measured broadband power elevations associated with perception, movement, language, and cognition [6, 70–72] Examples of broadband field potentials from single ECoG electrodes in motor cortex (A), ventral temporal cortex (B), and primary visual cortex (C). The power increases relative to baseline span at least 50 to 200 Hz. Adapted from (A) [73]; (B) [6]; (C) [74]. (TIFF) [file pone.0193107.s008.tiff]
